# Unified fair federated learning for digital healthcare

**DOI:** 10.1016/j.patter.2023.100907

**Published:** 2023-12-28

**Authors:** Fengda Zhang, Zitao Shuai, Kun Kuang, Fei Wu, Yueting Zhuang, Jun Xiao

**Affiliations:** 1 Zhejiang University, 38 Zheda Road, Hangzhou 310058, Zhejiang, China

**Keywords:** federated learning, algorithmic fairness, digital healthcare

## Abstract

Federated learning (FL) is a promising approach for healthcare institutions to train high-quality medical models collaboratively while protecting sensitive data privacy. However, FL models encounter fairness issues at diverse levels, leading to performance disparities across different subpopulations. To address this, we propose Federated Learning with Unified Fairness Objective (FedUFO), a unified framework consolidating diverse fairness levels within FL. By leveraging distributionally robust optimization and a unified uncertainty set, it ensures consistent performance across all subpopulations and enhances the overall efficacy of FL in healthcare and other domains while maintaining accuracy levels comparable with those of existing methods. Our model was validated by applying it to four digital healthcare tasks using real-world datasets in federated settings. Our collaborative machine learning paradigm not only promotes artificial intelligence in digital healthcare but also fosters social equity by embodying fairness.

## Highlights

• We enabled fair federated model training among healthcare institutions
• Our framework unifies diverse levels of fairness in federated learning
• Our efficient solution flexibly balances utility and different levels of fairness
• We applied our solution to the four digital healthcare tasks in federated settings

## The bigger picture

Federated learning (FL) enables healthcare institutions to collaboratively train high-quality medical machine learning models without accessing private medical data. Traditional FL may yield an unfair model with a good overall performance but still perform poorly with underrepresented subgroups. Because of the varied performance of these models among patients from different racial or ethnic groups, or those with specific physical conditions, differences in the quality of treatment recommendations may consequently cause serious social inequality problems. Frameworks that improve the fairness of FL will be essential in the implementation of digital healthcare.

## Graphical abstract

**Figure:**
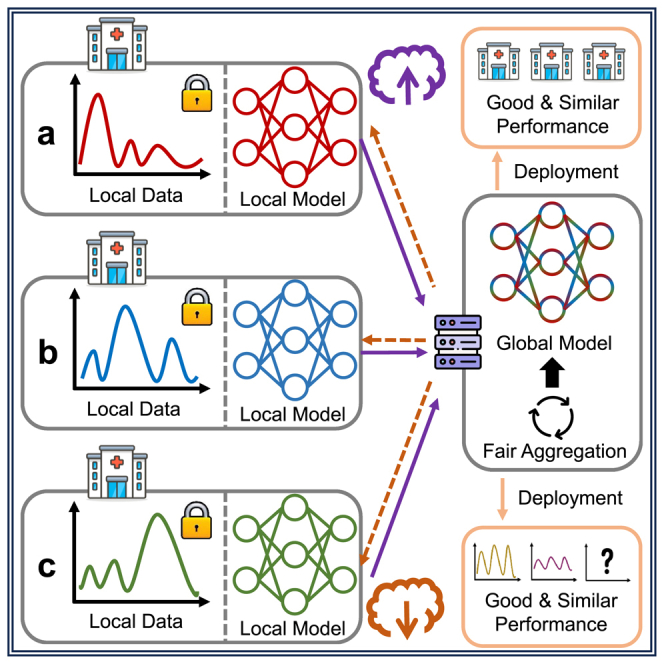

## Teaser

This study employed robust optimization to achieve consistent and high-performing federated models for diverse demographics. To ensure fairness in federated learning, a unified framework for healthcare institutions is presented that includes various fairness metrics and is implemented using an efficient and collaborative machine learning procedure. The proposed solution was tested in four digital medical scenarios in a federated setting. The experimental results show that the proposed approach could maintain model performance while improving the desired levels of fairness.

## Introduction

Artificial intelligence (AI) holds tremendous potential for revolutionizing the medical field and advancing digital health applications.^1^^,^^2^^,^^3^^,^^4^^,^^5^^,^^6^ However, its widespread implementation faces challenges, particularly concerning the handling of medical data and privacy concerns. Stringent regulations, such as the General Data Protection Regulation (GDPR) and Health Insurance Portability and Accountability Act (HIPAA), mandate the protection of sensitive patient information, making it impractical to gather all the necessary data for comprehensive AI training. Therefore, striking a delicate balance between harnessing the potential of AI and adhering to legal and ethical data privacy principles is important. To address this, researchers and developers must explore innovative approaches that enable AI models to be trained without compromising individual data privacy.^7^^,^^8^^,^^9^

Federated learning (FL) represents a crucial machine learning paradigm in which distributed clients (e.g., several medical institutions) collaboratively train a shared global model while retaining their private data.^10^^,^^11^^,^^12^^,^^13^ However, inherent biases may arise in the federated model because of spurious correlations and distribution shifts across data subpopulations.^14^^,^^15^^,^^16^^,^^17^^,^^18^ Consequently, the model’s performance may significantly degrade for certain data subpopulations, leading to concerns regarding unfairness, particularly in critical domains such as healthcare. Recently, addressing this issue and achieving an unbiased federated model with equitable performance have emerged as paramount objectives and pivotal research themes.

**Figure 1:**
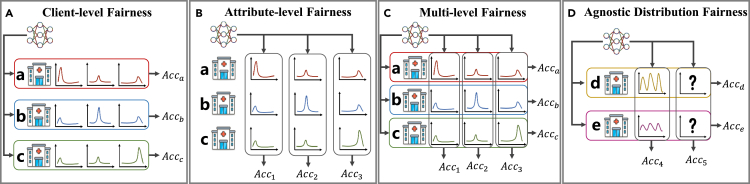
Illustration of diverse levels of fairness in federated learning scenario (A) Client-level fairness (horizontal fairness) requires that the federated model have consistent accuracy across different clients (hospitals), i.e., $Acc_{a}=Acc_{b}=Acc_{c}$. (B) Attribute-level fairness (vertical fairness) requires that the federated model have consistent accuracy across different attributes (e.g., physical conditions and/or sensitive demographics), i.e., $Acc_{1}=Acc_{2}=Acc_{3}$. (C) Multilevel fairness requires that the federated model achieve horizontal and vertical fairness simultaneously. (D) Agnostic distribution fairness requires that the federated model achieve fairness on subpopulations with unknown distributions (e.g., other hospitals that do not participate in FL), i.e., $Acc_{d}=Acc_{e}$ and $Acc_{4}=Acc_{5}$.

In this study, we present a systematic exploration of the multifaceted nature of the fairness of model performance in FL. To enhance its clarity, as shown in Figure 1, we categorize these levels into four distinct dimensions: client-level fairness, attribute-level fairness (also referred to as horizontal and vertical fairness, respectively), multilevel fairness, and agnostic distribution fairness, each with distinct practical implications for distribution fairness.

The primary objective for hospitals (clients) participating in FL is to obtain a model with optimal performance.^19^^,^^20^^,^^21^ The federated model must ensure that it does not disproportionately favor or disadvantage specific hospitals. Based on experience, a FL algorithm that neglects fairness considerations may yield a model of inferior quality compared with a model solely trained on local data. Consequently, hospitals experiencing poor performance in the federated model with their data may be discouraged from participating in FL initiatives. This reluctance could significantly hinder the development of a robust FL ecosystem and impede the broader application of AI in digital health.

Moreover, fairness at the attribute level, encompassing explicit covariates (e.g., gender and race), implicit groups (e.g., domain), the target variable, or their combinations, must also be ensured. For instance, in the context of a predictive model projecting a patient’s remaining lifespan, if the accuracy of the model significantly varies among patients of different races, it may lead to higher rates of incorrect treatments prescribed to certain racial groups, exacerbating social inequality.

A model violating any of the above fairness measures may lead to serious consequences; therefore, it is important to implement FL model performance fairness at multiple levels simultaneously, which is a more difficult problem than single-level fairness.

Finally, the generalizability of fairness in our medical machine learning model based on FL must be considered. This entails ensuring consistent performance not only within the existing participating hospitals but also in other hospitals with unseen distributions while maintaining fairness. By prioritizing this aspect, we can maximize social welfare in the medical field and ensure that our research findings have a meaningful impact on a broader scale.

Recently, progress in FL has led to increased research on achieving fair model performance.^22^ However, most of these studies have focused on single-level fairness, particularly at the client level.^23^^,^^24^^,^^25^^,^^26^^,^^27^^,^^28^^,^^29^^,^^30^ These methods can be broadly classified into two categories: personalization^31^^,^^32^^,^^33^ and fair aggregation.^34^ Personalization allows individual clients to maintain distinct local models, leveraging the diversity of other hospital data while emphasizing local data distribution. However, this approach fails to create a global model with robust generalization capabilities suitable for broad deployment. To overcome this limitation, the fair aggregation method assigns varying weights to local models of different clients during the aggregation process, ensuring consistent performance of aggregated models across diverse clients. To the best of our knowledge, while the aforementioned methods have contributed to the advancement of specific fairness in FL, limited attention has been given to unified and multilevel FL performance fairness.^35^^,^^36^^,^^37^

In this study, we attempted to find a solution for unified fairness in FL, with the requirement that federated models meet some or all of the above four levels of fairness. We also present a comprehensive framework called Federated Learning with Unified Fairness Objective (FedUFO). Our approach unifies the diverse levels of fairness considerations by leveraging a unified uncertainty set. Our unified framework considers existing optimization objectives as special cases, thereby providing a cohesive and encompassing perspective on fairness in FL. The model allows customizable uncertainty sets that offer users greater flexibility in managing the trade-offs between accuracy and fairness at various levels. Moreover, we address the challenging federated optimization problem by introducing a highly efficient algorithm called federated mirror descent ascent, which provides theoretical guarantees. Researchers and practitioners can gain a comprehensive understanding of performance fairness considerations in FL by adopting the proposed framework. This insight enables them to effectively assess and address fairness concerns, both coherently and meaningfully.

To validate the effectiveness of our approach, we conducted rigorous testing on four healthcare-related datasets and meticulously evaluated fairness at four distinct levels: client-level, attribute-level, multilevel, and agnostic distribution fairness. Our experimental findings clearly demonstrate that, in comparison with existing methods, our unified solution can improve fairness at the desired level(s) without any significant loss in overall accuracy. Moreover, our approach allows a flexible balance between accuracy and fairness, as well as between different fairness levels. These compelling results underscore our commitment to advancing fair and impactful FL practices with a dedicated focus on the crucial domain of medicine. Considering this, we aim to preserve the ecological integrity of FL in the medical field and actively promote social equality.

## Results

### Unified fair FL

#### Optimization objective for fair FL

We aim to encourage the federated model to achieve uniform performance over subpopulations. The unified optimization objective can be written as

$$R_{\text{fair}}\left(\theta \right)=\bar{R}\left(\theta \right)+C\sqrt{\frac{\text{Var}\left(R^{g}\left(\theta \right)\right)}{\left|G\right|}},$$

where $\bar{R}$ is the average risk, g∈G is the group index, $R^{g}$ is the risk of group *g*, $\text{Var}\left(R^{g}\left(\theta \right)\right)$ is the variance of risk across groups, and the constant C balances the utility and fairness. The first and second terms guarantee the utility of the federated model and the performance fairness for group G, respectively. $R_{\text{fair}}\left(\theta \right)$ is unified because G can be defined as any group as needed, and any single-level fairness in FL will be the special case of our framework.

#### Unified framework for fair FL

Unfortunately, $R_{\text{fair}}\left(\theta \right)$ is computationally intractable because of the variance term, particularly in a federated setting.^10^^,^^38^^,^^39^ To solve this problem, inspired by the techniques used for distributionally robust optimization (DRO),^40^ we introduced an approximate surrogate for $R_{\text{fair}}$:

$$R_{\text{dro}}\left(\theta \right)≔\underset{Q\in Q^{g}}{\sup}\left\{E_{\left(x,y\right)∼Q}\left[l\left(\theta ,\left(x,y\right)\right)\right]\right\},$$

and

$$Q^{g}≔\left\{\text{distribution}\;Q\;\text{such}\;\text{that}\;D_{f}\left(Q\|P^{G}\right)\leq \rho \right\},$$

where *ℓ*: $\Theta \times \left(X\times Y\right)\to R_{+}$ is a loss function, $P^{G}$ is the distribution when the entire data distribution is grouped based on G, $D_{f}\left(\cdot \|\cdot \right)$ is the *f*-divergence between distributions, ρ is the radius of the uncertainty set, and the uncertainty set $Q^{g}$ contains the distribution shifts near distribution $P^{G}$. The constant C and radius ρ are positively correlated; therefore, we can balance the model performance and fairness by varying the radius ρ. If the radius ρ=0, then $R_{\text{dro}}$ will degrade to empirical risk minimization (ERM) (FedAvg). Conversely, if we allow the radius to be infinite, then $R_{\text{dro}}$ will degrade to minimize the risk of the worst-performing group.

Next, we show that the optimization objectives designed for diverse levels of fairness are special cases of our proposed unified risk $R_{\text{dro}}$. First, if we select a client-level uncertainty set (i.e., G specified as client index set C), the unified objective will degrade to a client-level fairness method with a risk given by

$$R_{\text{client}}\left(\theta \right)≔\underset{Q\in Q^{c}}{\sup}\left\{E_{\left(x,y\right)∼Q}\left[l\left(\theta ,\left(x,y\right)\right)\right]\right\},$$

and

$$Q^{c}≔\left\{\text{distribution}\;Q\;\text{such}\;\text{that}\;D_{f}\left(Q\|P^{C}\right)\leq \rho \right\},$$

Similarly, if we select an attribute-level uncertainty set $Q^{a}$, the framework degrades to an attribute-level fairness FL method with a risk given by

$$R_{\text{attribute}}\left(\theta \right)≔\underset{Q\in Q^{a}}{\sup}\left\{E_{\left(x,y\right)∼Q}\left[l\left(\theta ,\left(x,y\right)\right)\right]\right\},$$

and

$$Q^{a}≔\left\{\text{distribution}\;Q\;\text{such}\;\text{that}\;D_{f}\left(Q\|P^{A}\right)\leq \rho \right\},$$

where $P^{A}$ is the distribution when all the data are grouped according to attribute index set A. Moreover, the corresponding objective that simultaneously constrains both the variance of client- and attribute-level risks is given below.

$$R_{\text{fair}}\left(\theta \right)=R\left(\theta \right)+C_{1}\sqrt{\frac{\text{Var}\left(R^{c}\left(\theta \right)\right)}{\left|C\right|}}+C_{2}\sqrt{\frac{\text{Var}\left(R^{a}\left(\theta \right)\right)}{\left|A\right|}}=\frac{C_{1}}{C_{1}+C_{2}}\left(R\left(\theta \right)+\left(C_{1}+C_{2}\right)\sqrt{\frac{\text{Var}\left(R^{c}\left(\theta \right)\right)}{\left|C\right|}}\right)+\frac{C_{2}}{C_{1}+C_{2}}\left(R\left(\theta \right)+\left(C_{1}+C_{2}\right)\sqrt{\frac{\text{Var}\left(R^{a}\left(\theta \right)\right)}{\left|A\right|}}\right)$$

This can be approximated by the following DRO-based objective:

$$R_{\text{multi}}\left(\theta \right)≔\underset{Q\in Q^{m}}{\sup}\left\{E_{\left(x,y\right)∼Q}\left[l\left(\theta ,\left(x,y\right)\right)\right]\right\},$$

and

$$Q^{m}≔\left\{\text{distribution}\;Q\;\text{such}\;\text{that}\;D_{f}\left(Q\left\|P^{C})\leq \beta \rho \;or\;D_{f}(Q\right\|P^{A}\right)\leq \left(1-\beta \right)\rho \right\},$$

where the uncertainty set of multilevel fairness is the union of the client-level and attribute-level uncertainty set; radius ρ balances the accuracy and the fairness; and the coefficient β balances different levels of fairness. Finally, we consider agnostic distribution fairness, which requires the federated model to be fair for subpopulations with unknown distributions. To achieve this goal, we defined a sufficiently wide uncertainty set that covers the possible distribution shifts. A natural implementation is to assign G as the combination of the client index and all the attributes, and then degrade the uncertainty set to the individual level, which is too wide by taking into account too much unnecessary distributional drift. Consequently, this may lead to an overly pessimistic problem in practice.^41^^,^^42^^,^^43^ Therefore, structural constraints must be introduced on the uncertainty set to overcome this pessimism. Moreover, we specified G as the combination of the client index C and some of the sensitive attribute(s) $\left\{A_{1},A_{2},\cdots \right\}$, rather than using all the attributes. The objective of the agnostic distribution fairness can be written as

$$R_{\text{unknown}}\left(\theta \right)≔\underset{Q\in Q^{u}}{\sup}\left\{E_{\left(x,y\right)∼Q}\left[l\left(\theta ,\left(x,y\right)\right)\right]\right\},$$

and

$$Q^{u}≔\left\{\text{distribution}\;Q\;\text{such}\;\text{that}\;D_{f}\left(Q\|P^{\left\{C,A_{1},A_{2},\cdots \right\}}\right)\leq \rho \right\},$$

Notably, a trade-off exists between in-distribution fairness and unknown out-of-distribution fairness because an overly conservative risk with a very wide uncertainty set usually leads to an upper bound that is too loose for in-distribution fairness. In practice, appropriate combinations can be used to balance the two. Please refer to Appendix B for more discussion on the size of uncertainty sets.

#### Tractable centralized optimization algorithm

Next, we developed an efficient algorithm to solve the above optimization objective $R_{\text{dro}}$ in federated setting. The objective $R_{\text{dro}}$ is rewritten as

$$\underset{\lambda^{g}\in \Delta_{\left|G\right|-1}}{\sup}\left\{F\left(\theta ,\lambda^{g}\right):=\sum_{i}\lambda_{i}^{g}f_{i}^{g}\left(\theta \right)\;s.t.\;D_{f}\left(\left|G\right|\cdot \lambda^{g}\|\left(1,1,\cdots ,1\right)\right)\leq \rho \right\},$$

where $f_{i}^{g}\left(\theta \right):=E_{\left(x,y\right)∼{\hat{P}}_{i}^{g}}\left[l\left(\theta ;\left(x,y\right)\right)\right]$ is the empirical risk on i th group and ${\hat{P}}_{i}^{g}$ is the empirical distribution over samples of data subset $D_{i}^{g}$. We can alternately optimize model parameters θ and weights $\lambda^{g}$ to minimize the above optimization objective. We updated model $\theta_{i}^{\left(t\right)}$ using the stochastic gradient descent method with corresponding weight ${\lambda_{i}^{g}}^{\left(t\right)}$ at each iteration t.

$$\theta_{i}^{\left(t+1\right)}=\theta_{i}^{\left(t\right)}-\eta {\lambda_{i}^{g}}^{\left(t\right)}\nabla_{\theta }l\left(\theta_{i}^{\left(t\right)};\left(x_{i}^{\left(t\right)},y_{i}^{\left(t\right)}\right)\right).$$

We adopted the mirror gradient ascent method to update weight $\lambda^{g}$, which is expressed as

$$\begin{array}{r} {\left({\tilde{\lambda }}^{g}\right)}^{\left(t+1\right)}={\mathrm{argmax}}_{\lambda \in \Delta_{\left|G\right|-1}}\{F\left(\theta^{\left(t+1\right)},{\left(\lambda^{g}\right)}^{\left(t\right)}\right)+\langle v^{\left(t\right)},{\left(\lambda^{g}\right)}^{\left(t\right)}-\lambda \rangle -\frac{1}{\gamma }D_{f}\left(\lambda ∥{\left(\lambda^{g}\right)}^{\left(t\right)}\right)\}, \end{array}$$

where γ>0 is the stepsize and $v^{\left(t\right)}$ is the gradient of weight. The first two terms are a linear approximation of $F\left(\theta^{\left(t+1\right)},\lambda \right)$, and the last term is a Bregman distance between λ and ${\left(\lambda^{g}\right)}^{\left(t\right)}$. A suitable convex function f(·) can be chosen to efficiently solve for ${\;\left({\tilde{\lambda }}^{g}\right)}^{\left(t+1\right)}$. By choosing the negative entropy function, ${\left({\tilde{\lambda }}^{g}\right)}^{\left(t+1\right)}$ has an explicit solution:

$${\left({\tilde{\lambda }}^{g}\right)}^{\left(t+1\right)}=\frac{{\left(\lambda_{i}^{g}\right)}^{\left(t\right)}e^{\gamma v_{i}^{\left(t\right)}}}{\sum_{i=1}^{\left|G\right|}{\left(\lambda_{i}^{g}\right)}^{\left(t\right)}e^{\gamma v_{i}^{\left(t\right)}}}.$$

After each mirror gradient ascent of weight, we compute ${\left(\lambda^{g}\right)}^{\left(t+1\right)}$ by projecting ${\left({\tilde{\lambda }}^{g}\right)}^{\left(t+1\right)}$ into $\left\{\lambda :D_{f}\left(\left|G\right|\cdot \lambda^{g}\|\left(1,1,\cdots ,1\right)\right)\leq \rho \right\}$ such that the constrains of the uncertainty set for the radius will be satisfied.^44^

We obtained the stochastic mirror descent ascent (SMDA) algorithm as the solution of objective $R_{\text{dro}}\left(\theta \right)$ in centralized setting, and we extended it to the decentralized setting.

#### Efficient federated optimization algorithm

In FL, each client accesses its own local data. In each communication, the clients use their local data to update local models with the corresponding weights, and then the server aggregates the local models into a global model. In addition, clients must compute the partial gradients of the weights and send them to the server for updating the weights. The key challenge is that high-frequency communication is not allowed in FL because of communication costs. Therefore, we adopted the snapshot mechanism for both the model and weight updates. Specifically, we updated the weights as follows:

$$\left\{{\left(\lambda_{i}^{g}\right)}^{\left(r+1\right)}\right\}=\text{Proj}\left(\left\{\frac{{\left(\lambda_{i}^{g}\right)}^{\left(r\right)}e^{\gamma E{v_{i}^{g}}^{\left(r\right)}}}{\sum_{i=1}^{\left|G\right|}{\left(\lambda_{i}^{g}\right)}^{\left(r\right)}e^{\gamma E{v_{i}^{g}}^{\left(r\right)}}}\right\},\rho \right),$$

where r is the current round of communication and E is the number of local iterations at each communication. The above equations can be viewed as an unbiased estimation of ${\left({\tilde{\lambda }}^{g}\right)}^{\left(t+1\right)}$. We allowed multiple iterations of the model parameters and weights in a single communication.

This completes the federated mirror descent ascent (FedUFO) algorithm. Taking multilevel fairness as an example, we present the details of FedUFO in Algorithm 1. Please refer to Appendix A for the proof of convergence of Algorithm 1.

Algorithm 1 **FedUFO algorithm for multilevel fairness**

**Input:** The number of local iterations E, total number of iterations T, number of rounds R=T/E, model update stepsize η, weight update stepsize γ, initialized model parameters $\theta^{\left(0\right)}$, client weight $\left\{{\lambda_{i}^{c}}^{\left(0\right)}\right\}$, attribute weight $\left\{{\lambda_{k}^{a}}^{\left(0\right)}\right\}$, uncertainty set radius ρ, and coefficient β∈[0,1] for balancing client and attribute-level fairness.

1: **for** r=0,1,…,R−1 **do**

2: The server broadcasts $\theta^{\left(rE\right)}$, $\left\{{\lambda_{i}^{c}}^{\left(r\right)}\right\}$, and $\left\{{\lambda_{k}^{a}}^{\left(r\right)}\right\}$ to the corresponding clients

3: **for** client i=1,2,…,N **do**

4:  Set the local model parameters $\theta_{i}^{\left(rE\right)}=\theta^{\left(rE\right)}$

5:  **for** t=rE,rE+1,…,(r+1)E−1 **do**

6:   Sample data $\xi_{i,k}^{\left(t\right)}$ uniformly

7: $\lambda_{i,k}^{\left(t\right)}={\lambda_{i}^{c}}^{\left(r\right)}$ *w*.*p*. β, and $\lambda_{i,k}^{\left(t\right)}={\lambda_{i}^{a}}^{\left(r\right)}$ *w*.*p*. 1−β

8:   Update the model with weights: $\theta_{i}^{\left(t+1\right)}=\theta_{i}^{\left(t\right)}-\eta {\lambda_{i,k}}^{\left(t\right)}\nabla l\left(\theta_{i}^{\left(t\right)};\xi_{i,k}^{\left(t\right)}\right)$

9:   Compute the loss ${v_{i}^{c}}^{\left(r\right)}$ of model $\theta^{\left(rE\right)}$ on the local dataset $D_{i}^{c}$

10:  Compute the loss ${v_{i,k}^{a}}^{\left(r\right)}$ of model $\theta^{\left(rE\right)}$ on each subgroup $D_{i,k}^{a}$

11:  **end for**

12: **end for**

13: The client i sends $\theta_{i}^{\left(\left(r+1\right)E\right)}$, ${v_{i}^{c}}^{\left(r\right)}$, and ${v_{i,k}^{a}}^{\left(r\right)}$ to the server

14: The server computes: $\theta^{\left(r+1\right)E}=\frac{1}{N}\sum_{i=1}^{N}\theta_{i}^{\left(\left(r+1\right)E\right)}$

15: The server computes: $\left\{{\left(\lambda_{i}^{c}\right)}^{\left(r+1\right)}\right\}=\text{Proj}\left(\left\{\frac{{\left(\lambda_{i}^{c}\right)}^{\left(r\right)}e^{\gamma E{v_{i}^{c}}^{\left(r\right)}}}{\sum_{i=1}^{N}{\left(\lambda_{i}^{c}\right)}^{\left(r\right)}e^{\gamma E{v_{i}^{c}}^{\left(r\right)}}}\right\},\rho \right)$

16: The server computes: $\left\{{\left(\lambda_{k}^{a}\right)}^{\left(r+1\right)}\right\}=\text{Proj}\left(\left\{\frac{{\left(\lambda_{k}^{a}\right)}^{\left(r\right)}e^{\gamma E\sum_{i=1}^{N}{v_{i,k}^{a}}^{\left(r\right)}}}{\sum_{k=1}^{M}{\left(\lambda_{k}^{a}\right)}^{\left(r\right)}e^{\gamma E\sum_{i=1}^{N}{v_{i,k}^{a}}^{\left(r\right)}}}\right\},\rho \right)$

17: **end for**

18: **return** $\theta^{\left(T\right)}$

**Figure 2:**
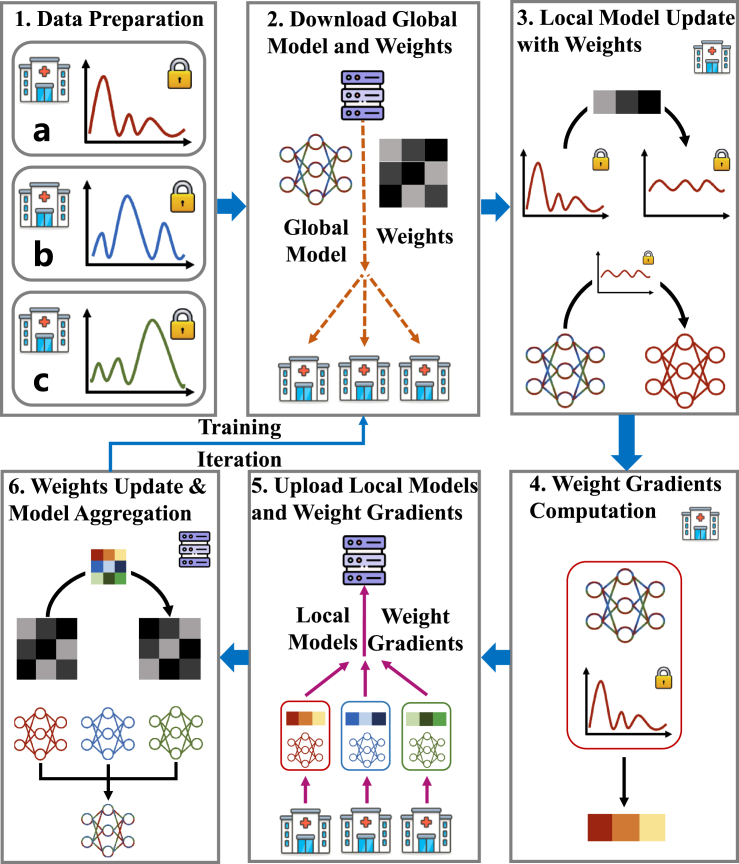
FedUFO training workflow Along with the conventional model aggregation process in standard federated learning, we incorporate an additional step to enhance model fairness by maintaining a dedicated set of weights. After training, the federated model with fairness guarantees can be deployed at different hospitals to assist in the medical diagnosis of patients belonging to different demographics.

An overview of the workflow is shown in Figure 2. In each communication, hospitals first download the global model and weights from the server and then update the local models with their private data with the corresponding weights. After computing the gradients of the weights, hospitals send them and the local models to the server. Then, the server aggregates the local models into a global model and updates the weights. These steps are repeated until the global model converges. The trained federated model can be deployed in various locations to aid in medical decisions. We emphasize that the proposed unified solution is general and can be specified to guarantee a given level of fairness. We would like to clarify here that our contributions focus on the diverse levels of fairness in FL, while the privacy protections of the proposed unified framework are entirely inherited from the FL paradigm itself.

### Main results

#### Evaluation metrics

Our aim is to guarantee that the model’s performance is good and fair across subpopulations. Suppose that the full dataset D is divided into |G| groups, $D=\left\{D_{1}^{g},D_{2}^{g},\ldots ,D_{\left|G\right|}^{g}\right\}$. We first define the disparity of an FL model across groups $\left\{D_{i}^{g}\left|i=1,2,\ldots \text{,}\right|G|\right\}$ as

$$Disparity=\sqrt{\frac{1}{\left|G\right|-1}\sum_{i=1}^{\left|G\right|}{\left(Acc\left(D_{i}^{g}\right)-Avg\_Acc\right)}^{2}},$$

where $Acc\left(D_{i}^{g}\right)$ is the predictive accuracy for group $D_{i}^{g}$ and $Avg\_Acc=\frac{1}{\left|G\right|}\sum_{i=1}^{\left|G\right|}Acc\left(D_{i}^{g}\right)$. In this study, following the difference principle of distributive justice and stability,^45^ we view the performance of the federated model as a resource that should be allocated fairly among the various groups. Specifically, we measured fairness using disparity. The smaller the value of disparity, the fairer the FL model. We focus on four levels of fairness in FL: client-level, attribute-level, multilevel, and agnostic distribution fairness. We measured client-level fairness using $\sqrt{\frac{1}{\left|C\right|-1}\sum_{i=1}^{\left|C\right|}{\left(Acc\left(D_{i}^{c}\right)-Avg\_Acc\right)}^{2}}$, where C is a set of client indices. Given a set of protected attributes A, the attribute-level fairness can be measured using $\sqrt{\frac{1}{\left|A\right|-1}\sum_{i=1}^{\left|A\right|}{\left(Acc\left(D_{i}^{a}\right)-Avg\_Acc\right)}^{2}}$. For multilevel fairness, we used the harmonic average of client-level fairness and attribute-level fairness as evaluation metrics. Notably, the harmonic average is just one of the indicators used to measure fairness at multiple levels. Different fairness metrics for various levels are presented in detail in Tables S1 and S2. Following the previous studies,^46^^,^^47^^,^^48^ we used a Dirichlet distribution with hyperparameter α to define data heterogeneity among different clients in FL. A lower value of α means strong data heterogeneity, and we set different values of α for different datasets to evaluate how our method performs at different degrees of client drift. For agnostic distribution fairness, we first trained a federated model with a specific value of α and then performed a simulation to test its multilevel fairness in federated settings for different values of α. Additionally, we used the accuracy, *Acc*, to measure the utility of the FL model. As part of our main results, we report the overall and worst-case performances for different scenarios.

#### Client-level fairness

The experimental results for client-level fairness are presented in Tables 1 and 2, respectively. We drew several conclusions based on these results. First, compared with local training (without [w.o.] FL) on the COVID-19 dataset, our solution decreased the disparity over clients from 0.0109 to 0.0010 (i.e., the fairness was improved by $\frac{0.0109-0.0010}{0.0109}=90.83\%$). Similarly, our solution, FedUFO, also improved fairness by 75.00%, 56.17%, and 83.75% for the fetal, prostate, and support datasets, respectively. Second, by comparing the federated baselines, we achieved a state-of-the-art performance. Taking the results for the support dataset as an example, the traditional federated algorithm FedAvg yields an unfair federated model with a disparity of 0.1026. Moreover, its accuracies for two hospitals were found to be 68.80% and 54.29%, indicating that the model was 14.51% less accurate for one hospital than the other. Our solution, FedUFO, limits the gap to 0.92% and significantly promotes fairness among hospitals. Third, compared with all the baselines, our client-level solution improved the worst client performance when tested on four different datasets, as shown in Table 2. Particularly, the accuracy of the proposed FedUFO improved by more than 10% compared with FedAvg in terms of the worst-performing client. In conclusion, our proposed FedUFO significantly improves client-level fairness, which helps prevent some hospitals from deploying federated models that perform poorly for their data distribution. Furthermore, this will encourage more hospitals to participate in FL and promote its wider application in the field of digital health.

**Table 1:** Client-level fairness: Disparity over clients^a^

Dataset	Heterogeneity	w.o. FL	Federated baselines					Ours	Centralized
	α	Local	FedAvg	AFL	q-FedAvg	FairFed^b^	Poulain’s^b^	FedUFO^c^	Global
Fetal	10,000	0.0324	0.0205	0.0121	0.0245	–	–	0.0081^c^	0.0288
Prostate	10	0.0162	0.0240	0.0397	0.0206	–	–	0.0071^c^	0.0199
COVID-19	0.5	0.0109	0.0306	0.0122	0.0194	0.0144	0.0114	0.0010^c^	0.0120
Support	0.1	0.0400	0.1026	0.0251	0.0121	0.0913	0.0339	0.0065^c^	0.0275

a Lower numbers are better.

b The federated baselines, FairFed and Poulain’s, only support settings of binary classification.

c Best federated learning results.

**Table 2:** Worst-case performance in client-level fairness: *Acc* for the worst client^a^ (%)

Dataset	Heterogeneity	w.o. FL	Federated baselines					Ours	Centralized
	α	Local	FedAvg	AFL	q-FedAvg	FairFed^b^	Poulain’s^b^	FedUFO_*c*_	Global
Fetal	10,000	87.72	95.32	94.15	92.98	–	–	96.49^c^	94.74
Prostate	10	71.04	79.11	77.41	79.22	–	–	79.98^c^	83.93
COVID-19	0.5	58.12	62.85	62.15	62.15	61.58	62.01	64.12^c^	64.97
Support	0.1	57.14	54.29	62.86	64.00	54.29	60.00	64.80^c^	65.71

a Higher numbers are better.

b The federated baselines, FairFed and Poulain’s, only support settings of binary classification.

c Best federated learning results.

#### Attribute-level fairness

The experimental results for attribute-level fairness are presented in Tables 3 and 4. An attribute can be specified as any variable (e.g., target variable, sensitive attribute, and their combinations). We chose the target variable, death or not, as the attribute for the COVID-19 dataset, and we expect that the federated model will satisfy the accuracy parity (AP) (i.e., similar false positives and false negatives). However, the false-negative and false-positive rates of the federated model trained by FedAvg were 82.67% and 45.89%, respectively (with disparity of 0.2601). The above results indicate that the federated model trained by FedAvg predicts that patients will not die, as the false-negative rate was 36.78% higher than the false-positive rate. If such a model is deployed on a large scale, many patients will die because of the lack of timely treatment, which will lead to serious social problems. Fortunately, the proposed solution can reduce the accuracy gap by less than 3% and, therefore, promote social fairness. Our algorithm, FedUFO, also achieved the lowest disparity and highest *Acc* of the worst attributes for the other datasets, demonstrating the effectiveness of our solution. Moreover, FedUFO can also avoid discrimination against specific races or genders. We present these results and discuss them in detail later.

**Table 3:** Attribute-level fairness: Disparity over attributes^a^

Dataset	Heterogeneity	w.o. FL	Federated baselines					Ours	Centralized
	α	Local	FedAvg	AFL	q-FedAvg	FairFed^b^	Poulain’s^b^	FedUFO_*a*_	Global
Fetal	10,000	0.1316	0.0778	0.0752	0.0643	–	–	0.0552^c^	0.0591
Prostate	10	0.3040	0.2420	0.2031	0.3175	–	–	0.1192^c^	0.2303
COVID-19	0.5	0.4716	0.2601	0.0660	0.1549	0.0236	0.0194	0.0174^c^	0.3424
Support	0.1	0.0487	0.1078	0.1235	0.0905	0.0637	0.0320	0.0134^c^	0.0349

a Lower numbers are better.

b The federated baselines, FairFed and Poulain’s, only support settings of binary classification.

c Best federated learning results.

**Table 4:** Worst-case performance in attribute-level fairness: *Acc* for the worst attribute^a^ (%)

Dataset	Heterogeneity	w.o. FL	Federated baselines					Ours	Centralized
	α	Local	FedAvg	AFL	q-FedAvg	FairFed^b^	Poulain’s^b^	FedUFO_*a*_	Global
Fetal	10,000	62.50	75.00	78.57	81.25	–	–	86.49^c^	83.33
Prostate	10	20.89	17.71	31.49	18.55	–	–	56.56^c^	19.37
COVID-19	0.5	25.54	45.89	58.12	52.10	60.65	61.43	62.52^c^	41.52
Support	0.1	57.89	60.19	54.39	56.14	61.17	62.14	63.16^c^	66.99

a Higher numbers are better.

b The federated baselines, FairFed and Poulain’s, only support settings of binary classification.

c Best federated learning results.

#### Multilevel fairness

In practice, breaches at any level of fairness can raise serious ethical issues; therefore, the federated model must be encouraged to be fair at multiple levels simultaneously. Specifically, we considered both client-level fairness and attribute-level fairness and used their harmonic average as the evaluation metric for multilevel fairness. Tables 5 and 6 list the experimental results for multilevel fairness. More detailed experimental results (client- and attribute-level metrics) are provided in the supplemental information. We can observe that FedUFO achieves the best FL results for all datasets. Notably, our solution is flexible because we allow users to balance the measure for multiple levels of fairness according to their needs by setting a hyperparameter. We present the results of the trade-offs in a later section.

**Table 5:** Multilevel fairness: Harmonic average^a^ of disparity over clients and disparity over attributes^b^

Dataset	Heterogeneity	w.o. FL	Federated baselines					Ours	Centralized
	α	Local	FedAvg	AFL	q-FedAvg	FairFed^c^	Poulain’s^c^	FedUFO_*m*_	Global
Fetal	10,000	0.0260	0.0162	0.0104	0.0177	–	–	0.0003^d^	0.0194
Prostate	10	0.0154	0.0218	0.0332	0.0193	–	–	0.0136^d^	0.0183
COVID-19	0.5	0.0107	0.0274	0.0103	0.0172	0.0089	0.0072	0.0016^d^	0.0116
Support	0.1	0.0220	0.0526	0.0209	0.0107	0.0375	0.0292	0.0021^d^	0.0154

a Disparity over clients and disparity over attributes are shown in the supplemental information (Table S1).

b Lower numbers are better.

c The federated baselines, FairFed and Poulain’s, only support settings of binary classification.

d Best federated learning results.

**Table 6:** Worst-case in multilevel fairness: Harmonic average^a^ of Acc for the worst client and Acc for the worst attribute^b^ (%)

Dataset	Heterogeneity	w.o. FL	Federated baselines					Ours	Centralized
	α	Local	FedAvg	AFL	q-FedAvg	FairFed^c^	Poulain’s^c^	FedUFO_*m*_	Global
Fetal	10,000	72.99	83.95	85.66	86.72	−	−	91.76^d^	88.67
Prostate	10	32.29	28.94	44.77	30.06	−	−	64.84^d^	31.48
COVID-19	0.5	35.49	53.05	60.07	56.68	61.11	61.72	63.17^d^	50.66
Support	0.1	57.51	57.09	58.32	59.81	57.53	61.05	62.50^d^	66.34

a *Acc* for the worst client and *Acc* for the worst attribute are shown in the supplemental information (Table S1).

b Higher numbers are better.

c The federated baselines, FairFed and Poulain’s, only support settings of binary classification.

d Best federated learning results.

#### Agnostic distribution fairness

To maximize social and medical benefits, powerful models derived from FL should be widely applicable. Therefore, the model should be deployable in hospitals that do not participate in FL and whose data have an unknown distribution. The experimental results for agnostic distribution fairness are listed in Tables 7 and 8. We set the heterogeneity hyperparameter α to 10, 5, and 2 during training and tested the trained federated model using α=1 by simulating a variety of differences between training environments and deployment environments. Although the performance of other methods greatly fluctuates with the degree of heterogeneity, our solution shows strong stability. Empirically, the results illustrate that FedUFO can effectively improve the fairness and worst-case performance (in terms of the harmonic average) of federated models, even if the target distributions are unknown.

**Table 7:** Agnostic distribution fairness: Harmonic average^a^ of Disparity over clients and Disparity over attributes^b^

Dataset	Heterogeneity^c^	w.o. FL	Federated baselines			Ours	Centralized
	α	Local	FedAvg	AFL	q-FedAvg	FedUFO_*u*_	Global
	10	0.0604	0.0568	0.0507	0.0495	0.0423^d^	0.0333
Prostate	5	0.0567	0.0676	0.0609	0.0668	0.0487^d^	0.0566
	2	0.0543	0.0556	0.0455	0.0450	0.0415^d^	0.0379

a Disparity over clients and disparity over attributes are shown in the supplemental information (Table S2).

b Lower numbers are better.

c We trained the federated models under various degrees of heterogeneity (including α = 10, 5, and 2) and evaluated the models for α = 1.

d Best federated learning results.

**Table 8:** Worst-case performance in agnostic distribution fairness: Harmonic average^a^ of *Acc* for the worst client and *Acc* for the worst attribute^b^

Dataset	Heterogeneity^c^	w.o. FL	Federated baselines			Ours	Centralized
	α	Local	FedAvg	AFL	q-FedAvg	FedUFO_*u*_	Global
	10	33.49	50.99	55.57	49.87	67.18^d^	47.14
Prostate	5	40.71	57.11	43.66	59.86	66.85^d^	63.81
	2	36.50	55.28	58.76	58.59	68.77^d^	54.57

a *Acc* for the worst client and *Acc* for the worst attribute are shown in the supplemental information (Table S2).

b Higher numbers are better.

c We trained the federated models under various degrees of heterogeneity (including α = 10, 5, and 2) and evaluated the models for α = 1.

d Best federated learning results.

#### Model utility

A fair but poorly performing model is meaningless.^49^ For example, if a model has an accuracy of zero for all groups, then the model meets the fairness requirement but does not contribute to medical decisions. Therefore, we also focused on model utility measured by overall accuracy (in terms of macro-averaged performance weighted by sample size). Our solutions achieved FL results comparable with the standard FL algorithm FedAvg (without consideration of fairness), as shown in Table 9.

**Table 9:** Overall performance: Macro-averaged accuracy^a^ (%)

Dataset	Heterogeneity	w.o. FL	Federated baselines					Ours				Centralized
	α	Local	FedAvg	AFL	q-FedAvg	FairFed^b^	Poulain’s^b^	FedUFO_*c*_	FedUFO_*a*_	FedUFO_*m*_	FedUFO_*u*_	Global
Fetal	10,000	90.00	96.76	95.00	94.71	–	–	97.06	94.71	96.47	95.41	96.76
Prostate	10	72.18	80.74	79.48	80.29	–	–	80.46	80.48	80.60	80.19	85.28
COVID-19	0.5	58.67	64.40	62.76	63.12	62.31	62.58	64.21	63.76	63.49	63.21	65.58
Support	0.1	61.56	65.62	65.62	64.37	64.37	63.75	65.00	64.38	63.13	64.28	68.75

a Higher numbers are better.

b The federated baselines, FairFed and Poulain’s, only support settings of binary classification.

### Trade-off analysis

**Figure 3:**
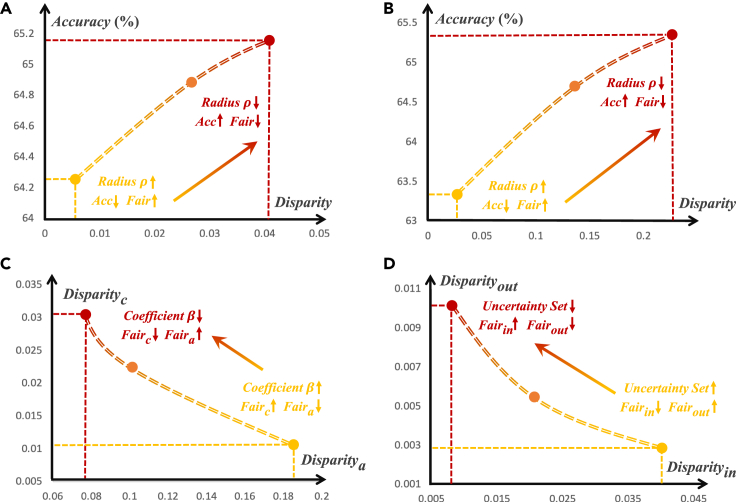
Trade-offs experimental results on COVID-19 dataset Heterogeneity α = 0.1. Our solution FedUFO is flexible and can balance different goals. (A) Trade-off between client-level fairness and accuracy. (B) Trade-off between attribute-level fairness and accuracy. (C) Trade-off between client-level fairness and attribute-level fairness. (D) Trade-off between in-distribution fairness and out-of-distribution fairness.

The general framework we proposed can not only constrain any given level(s) of fairness in FL but also allow users the flexibility to make trade-offs among different goals based on their own needs. Specifically, users can balance (1) accuracy and fairness, (2) multiple levels of fairness, and (3) in-distribution fairness and out-of-distribution fairness. We ran the analytical experiments on the COVID-19 dataset with a heterogeneity of α = 0.1, and the experimental results are shown in Figure 3.

#### Trade-off between accuracy and single-level fairness

First, we can balance the model’s utility and fairness via the uncertainty set radius ρ. An uncertainty set with a larger radius ρ considers more potential distribution shifts; thus, it can provide a fairness guarantee for the worst cases. However, this also places a looser upper bound on the ERM, leading to a decrease in accuracy.

As shown in Figures 3A and 3B, when we decrease the radius ρ from 1e−1 to 1e−8, the disparity among clients and attributes increases while the accuracy is improved.

#### Trade-off between multiple levels of fairness

We also set a hyperparameter β∈[0,1] to balance client-level and attribute-level fairness. We varied the value of β from 0.1 to 0.9, as shown in Figure 3C, and found that client-level fairness benefits from a large coefficient β, whereas attribute-level fairness benefits from a small β.

#### Trade-off between in-distribution fairness and out-of-distribution distribution fairness

A wide uncertainty set can be used to improve the generalization ability of fairness. However, an overly wide uncertainty set results in overly pessimistic problems, resulting in a lot of accuracy sacrifice. Empirically, we considered three uncertainty sets of different sizes formed by the client index, attribute, and uncertainty set radius. For a small uncertainty set, we used the union of the client-level uncertainty set and attribute-level uncertainty set with β=0.5. For the two large uncertainty sets, formed by the combination of client index and target variable, we set the uncertainty set radius ρ as 1e−7 and 1e−1, respectively. The large uncertainty set yields a model with good out-of-distribution fairness but compromises in-distribution fairness. The empirical evidence supports our analysis, as shown in Figure 3D. We recommend the construction of an uncertainty set based on specific requirements and expert knowledge.

We wish to elucidate that the flexibility extended here aims to offer users a realm of choice, enabling them to judiciously forego certain indicators of lesser importance to them in favor of enhancing those they deem significant, based on their actual needs. The extent of alterations in the ultimate outcome of the indicator is, to a degree, shaped by the pre-set data distribution.

### Relation with other fairness notions

In this study, our aim was to encourage the federated model to have a low accuracy disparity among different groups. The definition of fairness in this paper is different from traditional algorithmic fairness, which requires the independence of model decisions and sensitive attributes.^47^^,^^50^^,^^51^^,^^52^^,^^53^^,^^54^^,^^55^^,^^56^ However, we demonstrated that our proposed unified fairness notions can also be used to improve algorithmic fairness. Specifically, equal opportunity (EO),^57^ the most commonly used metric for measuring fairness (in terms of discrimination against certain groups), can be viewed as a relaxed version of fairness. The EO requires the following:

$$P\left(Y=1|\hat{Y}=1,A=0\right)=P\left(Y=1|\hat{Y}=1,A=1\right),$$

If we specify *S* as a combination of the target label and sensitive attribute, our proposed unified fairness notion requires that

$$P\left(Y=\hat{Y}|\hat{Y}=0,A=0\right)=P\left(Y=\hat{Y}|\hat{Y}=0,A=1\right)=P\left(Y=\hat{Y}|\hat{Y}=1,A=0\right)=P\left(Y=\hat{Y}|\hat{Y}=1,A=1\right),$$

Therefore, our optimization objective provides an upper bound for EO.

We empirically evaluated the models trained using different algorithms in terms of EO. The sensitive attribute was race, and the target variable was treatment outcome. FairFed^47^ and Poulain’s FairFedAvg^48^ are two state-of-the-art methods that aim to mitigate discrimination (in terms of sensitive attributes) in FL. Following the setting in the previous work,^48^ we set the heterogeneity hyperparameter α = 1. We also report the worst-case performance, which was measured using the following metrics:

$$\text{WorstTPR}=\min_{n}\;\mathrm{Pr}\left(\hat{Y}=1|A=n,Y=1\right)\text{.},$$

The experimental results are listed in Table 10. We can observe that our solution, FedUFO, achieves the best results in terms of EO and WorstTPR, while maintaining comparable overall performance with the standard FL algorithm, FedAvg (without consideration of fairness). These results illustrate that the fairness framework is unified and flexible.

**Table 10:** Federated results of discrimination against specific sensitive attribute

Dataset	Metrics	w.o. FL	Federated baselines					Ours	Centralized
		Local	FedAvg	AFL	q-FedAvg	FairFed	Poulain’s	FedUFO_*a*_	Global
COVID-19	EO^a^	0.300	0.318	0.267	0.299	0.211	0.174	0.160^d^	0.265
	WorstTPR^b^ (%)	12.17	36.12	28.52	19.39	49.80	47.15	61.60^d^	39.92
	Overall *Acc*^c^ (%)	57.49	62.34^d^	59.17	58.89	59.34	59.17	61.52	66.24

a Lower numbers are better.

b Higher numbers are better.

c Higher numbers are better.

d Best federated learning results.

We recommend setting *S* on the basis of expert knowledge in practice.

### Sensitivity analysis for FL

Our method is robust to changes in the hyperparameters of the FL settings. The general framework of our method is not sensitive to variations in the number of clients or local iterations. In this section, we modify these two hyperparameters and test the performance of our model on a fetal dataset.

#### Number of clients N

**Figure 4:**
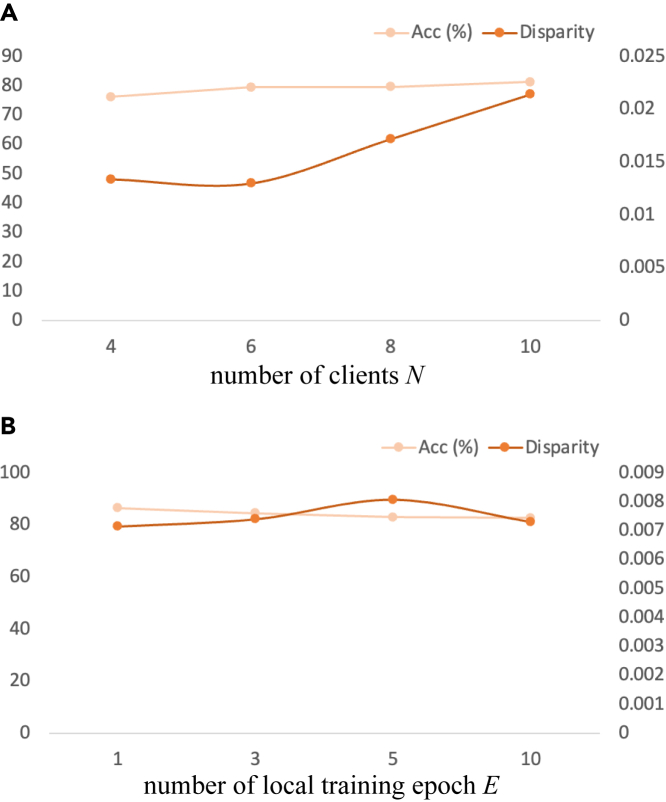
Sensitivity analysis experimental results (A and B) Our solution, FedUFO, is robust to variations in the number of clients N and the number of local iterations E.

Our model was robust to the change in the number of clients. We set the heterogeneity hyperparameter α of the dataset to 10 and the number of clients to 4, 6, 8, and 10 and recorded the variation in accuracy and fairness to investigate the sensitivity to the change in the number of clients in our model. As shown in Figure 4A, the accuracy of our model fluctuates only slightly, within a range of 5%, around 79%, and the fairness of our model holds up to 0.013 when the number increases from four to six.

#### Number of local iteration epochs E

Our model is insensitive to the number of local iterations. We set the heterogeneity hyperparameter α of the dataset to 1 and changed the number of iterations to 1, 3, 5, and 10 to verify the stability of the performance of our model. In Figure 4B, the range of accuracy is approximately 3% and that of fairness is less than 0.001, indicating that our model maintains high performance and a high level of fairness despite variation in the number of local iterations.

## Discussion

This study investigated fairness considerations in the realm of FL, particularly within the domain of digital healthcare. Our aim was to establish a consistent performance standard across various subpopulations using the federated model. To achieve this, we developed a unified framework for fair FL that offers adaptability for enhancing fairness at multiple levels, ranging from client- and attribute-level fairness to multilevel fairness and fairness extensions to uncharted data distributions, based on user requirements. In addition, we introduced an efficient optimization algorithm tailored for FL, which is essential for implementing the aforementioned framework and has been substantiated by comprehensive theoretical analyses.

We conducted extensive experimentation on four real-world medical datasets, encompassing a spectrum of FL scenarios. When compared with a diverse array of advanced federated baseline methodologies, our approach consistently demonstrated superior fairness outcomes at the desired fairness levels in most instances. Additionally, we elucidated the adaptability inherent in our framework through experimental scrutiny, showcasing its capacity to enable models to strike an optimal balance between fairness and accuracy. This adaptability also permits trade-offs in fairness levels across varying strata.

Moreover, our investigation addresses the issue of potential bias or discrimination arising from federated models. Theoretically, we posit that mitigating such concerns can be approached as a sub-problem inherent in the broader, unified framework that we propose. Empirical assessments demonstrated the effectiveness of our approach in mitigating discrimination, accomplished through the prudent selection of uncertainty set ranges while maintaining model performance comparable with the standard FL benchmark, FedAvg.

However, the proposed approach has certain limitations. For instance, the precise determination of uncertainty set ranges prior to model training poses a challenge, despite its pronounced influence on model outcomes. In practical applications, we recommend that users integrate domain expertise with predetermined hyperparameter settings for the uncertainty set and test the parameters on a limited subset of data before embarking on large-scale training. We expect that further investigation into determination of the uncertainty set will be conducted in future studies.

## Experimental procedures

### Resource availability

#### Lead contact

Further information, questions, and requests should be sent to Kun Kuang (kunkuang@zju.edu.cn).

#### Materials availability

This study did not involve any physical materials.

#### Data and code availability

Our source code is available at GitHub (https://github.com/Zitao-Shuai/FedUFO) and has been archived at Zenodo.^58^

### Datasets

We conducted our experiments on four medical datasets: (1) prostate cancer datasets from the US (prostate),^59^ (2) a fetal state dataset of cardiotocography (fetal),^60^ (3) a COVID-19 dataset of Brazilian patients (COVID-19);^61^ and (4) a support dataset of seriously ill hospitalized adults (support).^62^

All the datasets were tabular and were used for classification. (1) The prostate dataset has been widely used for forecasting tumor types. We selected features, such as age at diagnosis, race, sex, year of diagnosis, site, morphology group, and therapy group, in the period 2017–2020, from the Surveillance, Epidemiology, and End Results (SEER) database. For data preprocessing, we used the site recode ICD-O-3/WHO 2008 as the target variable. We recoded the top nine attributes based on the number of samples as nine classes and recoded the remaining attributes as the tenth class. Therefore, we constructed a 10-classification task. Then, we transformed the other input features into the one-hot coding form and dropped the data records with null value. Ultimately, 287,237 data records and 66 features were obtained. For the remaining datasets, we followed the method of data processing described by Seedat et al.^63^ and dropped the rows with null values. (2) The fetal cardio dataset is a 10-classification dataset used to identify cardiovascular diseases. Ultimately, 2,123 data records and 35 features were obtained. (3) The COVID-19 dataset is a binary classification dataset used to analyze the relationship between several factors and deaths caused by COVID-19.^64^ For this dataset, 6,882 data records and 44 features were obtained. (4) The support dataset is another popular dataset used in digital medical analyses. For this dataset, we obtained 1,000 data records and 30 features.

The fairness of algorithms for sensitive attributes (e.g., age, sex, and race) is an important concern. In this study, we took sex as an example and ran comprehensive experiments on the prostate and COVID-19 datasets to evaluate the performance of different algorithms in terms of fairness.

For training and evaluation, the dataset was randomly divided into two parts. The first part accounted for 20% of the total dataset and was used to evaluate the equalized opportunity of the models. The second part was used as the primary dataset for subsequent experiments. Given *N* clients, we used the Latent Dirichlet Allocation (LDA)^65^^,^^66^ algorithm to divide the second part into *N* parts for each client. The non-IID (independent and identically distributed) degree of these datasets was controlled by the hyperparameter α of the algorithm. For each client, we randomly divided the dataset into sets with 80% and 20% of the data. The set with 80% of the data was used as the training set, and the set with 20% of the data was used for testing.

The detailed breakdown of the distribution of each dataset is shown in Table 11.

**Table 11:** Detailed breakdown of the distribution of datasets

Fetal state dataset of cardiotocography: Training distribution											
α = 10,000		Attr 1	Attr 2	Attr 3	Attr 4	Attr 5	Attr 6	Attr 7	Attr 8	Attr 9	Attr 10
Client 1		126	179	17	24	20	109	80	35	23	63
Client 2		122	193	19	26	22	106	81	31	21	63

**Fetal state dataset of cardiotocography: Test distribution**											

α = 10,000		Attr 1	Attr 2	Attr 3	Attr 4	Attr 5	Attr 6	Attr 7	Attr 8	Attr 9	Attr 10
Client 1		27	53	5	8	6	24	18	6	3	19
Client 2		31	40	5	6	6	30	19	10	5	19

**Prostate cancer datasets from the US: Training distribution**											

α = 10	Attr 1	Attr 2	Attr 3	Attr 4	Attr 5	Attr 6	Attr 7	Attr 8	Attr 9	Attr 10	Attr 11
Client 1	23,632	7,288	5,697	5,265	6,127	5,074	3,673	3,809	3,021	3,270	28,575
Client 2	31,458	6,305	5,549	5,855	3,280	2,777	2,329	1,753	2,351	1,306	25,437

**Prostate cancer datasets from the US: Test distribution**											

α = 10	Attr 1	Attr 2	Attr 3	Attr 4	Attr 5	Attr 6	Attr 7	Attr 8	Attr 9	Attr 10	Attr 11
Client 1	5,789	1,847	1,438	1,375	1,507	1,274	941	1,009	782	820	7,076
Client 2	7,740	1,690	1,380	1,405	846	722	575	392	607	344	6,399
COVID-19 dataset: Training distribution						COVID-19 dataset: Test distribution					
α = 0.5		Attr 1		Attr 2		α = 0.5		Attr 1		Attr 2	
Client 1		1,500		1,332		Client 1		359		349	
Client 2		834		738		Client 2		195		198	
Support dataset: Training distribution						Support dataset: Test distribution					
α = 0.1		Attr 1		Attr 2		α = 0.1		Attr 1		Attr 2	
Client 1		171		329		Client 1		49		76	
Client 2		35		105		Client 2		8		27	

### Compared methods

We used the following baselines as our comparison methods: FedAvg,^10^ AFL,^24^ q-FedAvg,^23^ FairFed,^47^ and Poulain’s FairFedAvg.^48^ FedAvg is a classic baseline of the FL domain that simply aggregates local models with equal weights. AFL and q-FedAvg are two popular FL methods aiming to alleviate unfairness at the client level, while FairFed^47^ and Poulain’s FairFedAvg^48^ are two state-of-the-art attribute-level methods. We also trained local models on the clients’ own training datasets (called local). Additionally, we trained a model on the union of the training dataset for each client in a centralized setting (called global).

### Experimental settings

#### Model structure and hyperparameter setting

For the smaller datasets, COVID-19, fetal, and support, we utilized a 64-dimension fully connected (FC) layer as our backbone network. We set the learning rate between 5e−1 and 5e−4 for the global method and 0.01 for the other methods, and the batch size to 32. The number of local training epochs, *E*, was set to five. For the Prostate dataset, we utilized a 256-dimensional FC layer as the backbone network. We set the learning rate of the global method to 5e−3 and 1e−3 for the other methods, and the batch size to 128. The number of epochs of the local iterations was set to two. The number of communications for each dataset was set to ten. All the methods shared the same network structure. We used the Adam^67^ algorithm as the optimizer in our experiments. For the main experiment, the γ value for all the methods ranged from 1 to 10 for the prostate and support datasets and from 1e−6 to 1e−2 for the fetal and COVID-19 datasets. In the main experiment, the β value used to measure the attribute-level and client-level fairness was set to 5e−1, and the radius was set to 1e−4.

### Model selection and evaluation

For each method, we restored the model at each checkpoint after five communications and selected the model for evaluation based on its total loss in all training datasets. We used the standard deviation of the accuracy of model on different clients to measure client-level fairness and the standard deviation of the accuracy of model on different attributes to measure attribute-level fairness. For multilevel fairness, we used the harmonic average of client-level fairness and attribute-level fairness as the evaluation metric. To measure agnostic fairness, we split a given dataset into two different non-independent and identically distributed (i.i.d.) degrees. First, we trained the models under one of the partitions in a federated setting and then evaluated the aggregated model for the other partition.

### Supplemental information

Appendix A presents the convergence rates of the proposed FedUFO algorithm and its theoretical guarantees. In Appendix B, we discuss the effect of the size of the uncertainty set in FedUFO on the fairness and performance of the federated model, showing why we used an uncertainty set that is considerably large and may lead to failure theoretically. In Appendix C, we provide additional experimental results for multilevel fairness (Table S1) and agnostic distribution fairness (Table S2) to demonstrate the effectiveness of our solution, FedUFO.

## Supplemental information

Document S1. Appendices A–C and Tables S1 and S2

Document S2. Article plus supplemental information
