# Supplementary material for: Unified fair federated learning for digital healthcare
Source: Patterns (N Y). 2023 Dec 28;5(1):100907. doi: 10.1016/j.patter.2023.100907 (PMC10801255; doi:10.1016/j.patter.2023.100907)
Supplement: Document S1. Appendices A–C and Tables S1 and S2 [file mmc1.pdf]

Patterns, Volume *5*

Supplemental information

Unified fair federated learning for digital healthcare

Fengda Zhang, Zitao Shuai, Kun Kuang, Fei Wu, Yueting Zhuang, and Jun Xiao

Appendix A. CONVERGENCE ANALYSIS

In this section, we determine the convergence rate of the proposed FedUFO algorithm.

Theorem 1. *Suppose that each function $f_{i,k}$ is convex and L-smooth, the global function F is linear in $\boldsymbol{\lambda}$ and L-smooth, and the gradient w.r.t. θ and λ, model parameters θ, and the variance of the stochastic gradient method w.r.t. θ and λ are bounded. If we optimize $\mathcal{R}_{\text{dro}}$ using FedUFO algorithm with local iterations $E = O\left(T^{\frac{1}{4}}\right)$, the learning rate for model parameters $\eta = O\left(T^{-\frac{1}{2}}\right)$, and stepsize for group weights $\gamma = O\left(T^{-\frac{1}{2}}\right)$, then $\varepsilon_T \leq O\left(T^{-\frac{1}{2}}\right)$ holds.*

We analyse the convergence rate of Algorithm 1 by bounding the error ε_T defined as

$$\varepsilon_T = \max_{\boldsymbol{\lambda}^u} \mathbb{E}\left[F\left(\overleftarrow{\theta}^{(T)}, \boldsymbol{\lambda}^u\right)\right] - \min_{\theta} \mathbb{E}\left[F\left(\theta, \overleftarrow{\boldsymbol{\lambda}^u}^{(T)}\right)\right],$$

where T is the number of total iterations, $\overleftarrow{\theta}^{(T)}$ is the average of global model parameters of T iterations, and $\overleftarrow{\boldsymbol{\lambda}^u}^{(T)}$ is the average of group weights of T iterations. First, we introduce several technical lemmas.

Lemma 2. *The stochastic gradient $\mathbf{u}^{(t)}$ is defined as*

$$\mathbf{u}^{(t)} := \frac{1}{K}\sum_{i \in U\left(\lfloor \frac{t}{E} \rfloor\right)} \nabla f_i\left(\theta_i^{(t)}; \xi_i^{(t)}\right) = \frac{1}{K}\sum_{i \in U\left(\lfloor \frac{t}{E} \rfloor\right)} \sum_{j=1}^{M_i} \frac{\lambda_{i,k}^{u\left(\lfloor \frac{t}{E} \rfloor\right)}}{\lambda_i^{c\left(\lfloor \frac{t}{E} \rfloor\right)}} \nabla f_{i,k}\left(\theta_i^{(t)}; \xi_i^{(t)}\right)$$

which is unbiased, and its variance is bounded, implying that

$$\begin{aligned}
&\mathbb{E}_{\xi_i^{(t)}, U\left(\lfloor \frac{t}{E} \rfloor\right)}\left[\frac{1}{K}\sum_{i \in U\left(\lfloor \frac{t}{E} \rfloor\right)} \sum_{j=1}^{M_i} \frac{\lambda_{i,k}^{g\left(\lfloor \frac{t}{E} \rfloor\right)}}{\lambda_i^{c\left(\lfloor \frac{t}{E} \rfloor\right)}} \nabla f_{i,k}\left(\theta_i^{(t)}; \xi_i^{(t)}\right)\right] \\
= \; &\mathbb{E}_{U\left(\lfloor \frac{t}{E} \rfloor\right)}\left[\overleftarrow{\mathbf{u}}^{(t)} := \frac{1}{K}\sum_{i \in U\left(\lfloor \frac{t}{E} \rfloor\right)} \sum_{j=1}^{M_i} \frac{\lambda_{i,k}^{u\left(\lfloor \frac{t}{E} \rfloor\right)}}{\lambda_i^{c\left(\lfloor \frac{t}{E} \rfloor\right)}} \nabla f_{i,k}\left(\theta_i^{(t)}\right)\right], \\
= \; &\mathbb{E}\left[\sum_{i=1}^{N}\sum_{j=1}^{M_i} \lambda_{i,k}^{g\left(\lfloor \frac{t}{E} \rfloor\right)} \nabla f_{i,k}\left(\theta_i^{(t)}\right)\right]
\end{aligned}$$

$$\mathbb{E}\left[\| \mathbf{u}^{(t)} - \overleftarrow{\mathbf{u}}^{(t)} \|_2^2\right] \leq \frac{B^2}{K},$$

where $B > 0$ is a constant bound.

Proof. The stochastic gradient $\boldsymbol{u}^{(t)}$ is unbiased because we sampled the groups based on $\boldsymbol{\lambda}^{u\left(\lfloor \frac{t}{E} \rfloor\right)}$. The variance term is based on the assumption of Theorem 1.

Inspired by Li et al.[S1], we introduce the gradient dissimilarity Γ, which is defined as

$$\Gamma := \sup_{\theta, \boldsymbol{p} \in \Delta_{N-1}, i \in [N]} \sum_{j=1}^{N} p_j \|\nabla f_i(\theta) - \nabla f_j(\theta)\|_2^2,$$

where $f_i(\theta)$ is the local objective of client i.

□

Lemma 3. *Define $\delta^{(t)} := \frac{1}{K}\sum_{i\in U^{(\lfloor \frac{t}{E} \rfloor)}} \| \theta_i^{(t)} - \theta^{(t)}\|_2^2$. For FedUFO, the expected average squared norm distance of local models $\theta_i^{(t)}, i \in U^{(\lfloor \frac{t}{E} \rfloor)}$, and $\theta^{(t)}$ is bounded as $\frac{1}{T}\sum_{t=0}^{T} \mathbb{E}\left[\delta^{(t)}\right] \leq 10\eta^2 E^2 \left(B^2 + \frac{B^2}{K} + \Gamma\right)$, where the expectation is taken over the sampling of devices at each iteration.*

Proof. Considering $rE \leq t \leq (r+1)E$, we have

$$\begin{aligned}
\mathbb{E}\left[\delta^{(t)}\right] = \;& \mathbb{E}\left[\frac{1}{K}\sum_{i\in U^{(\lfloor \frac{t}{E} \rfloor)}} \| \theta_i^{(t)} - \theta^{(t)} \|_2^2\right] \\
\leq \;& \mathbb{E}\left[\frac{1}{K}\sum_{i\in U^{(\lfloor \frac{t}{E} \rfloor)}} \mathbb{E}\, \|\theta^{(rE)} - \sum_{s=rE}^{t-1} \eta\, \nabla f_i\left(\theta_i^{(s)}; \xi_i^{(s)}\right) - \left(\theta^{(rE)} - \frac{1}{K}\sum_{i'\in U} \sum_{s=rE}^{t-1} \eta\, \nabla f_{i'}\left(\theta_{i'}^{(s)}; \xi_{i'}^{(s)}\right)\right) \|_2^2\right] \\
= \;& \mathbb{E}\left[\frac{1}{K}\sum_{i\in U^{(\lfloor \frac{t}{E} \rfloor)}} \| \sum_{s=rE}^{t-1} \eta\, \nabla f_i\left(\theta_i^{(s)}; \xi_i^{(s)}\right) - \frac{1}{K}\sum_{i'\in U^{(\lfloor \frac{t}{E} \rfloor)}} \sum_{s=rE}^{t-1} \eta\, \nabla f_{i'}\left(\theta_{i'}^{(s)}; \xi_{i'}^{(s)}\right) \|_2^2\right] \\
\leq \;& \mathbb{E}\left[\frac{1}{K}\sum_{i\in U^{(\lfloor \frac{t}{E} \rfloor)}} \eta^2\, E \sum_{s=rE}^{(r+1)E} \| \nabla f_i\left(\theta_i^{(s)}; \xi_i^{(s)}\right) - \frac{1}{K}\sum_{i'\in U^{(\lfloor \frac{t}{E} \rfloor)}} \nabla f_{i'}\left(\theta_{i'}^{(s)}; \xi_{i'}^{(s)}\right) \|_2^2\right] \\
= \;& \eta^2 E \mathbb{E}[\frac{1}{K}\sum_{i\in U^{(\lfloor \frac{t}{E} \rfloor)}} \sum_{s=rE}^{(r+1)E} \| \nabla f_i\left(\theta_i^{(s)}; \xi_i^{(s)}\right) - \nabla f_i\left(\theta_i^{(s)}\right) + \nabla f_i\left(\theta_i^{(s)}\right) - \nabla f_i\left(\theta^{(s)}\right) + \nabla f_i\left(\theta^{(s)}\right) \\
& - \frac{1}{K}\sum_{i'\in U^{(\lfloor \frac{t}{E} \rfloor)}} \nabla f_{i'}\left(\theta^{(s)}\right) + \frac{1}{K}\sum_{i'\in U^{(\lfloor \frac{t}{E} \rfloor)}} \nabla f_{i'}\left(\theta^{(s)}\right) - \frac{1}{K}\sum_{i'\in U^{(\lfloor \frac{t}{E} \rfloor)}} \nabla f_{i'}\left(\theta_{i'}^{(s)}\right) \\
& + \frac{1}{K}\sum_{i'\in U^{(\lfloor \frac{t}{E} \rfloor)}} \nabla f_{i'}\left(\theta_{i'}^{(s)}\right) - \frac{1}{K}\sum_{i'\in U^{(\lfloor \frac{t}{E} \rfloor)}} \nabla f_{i'}\left(\theta_{i'}^{(s)}; \xi_{i'}^{(s)}\right) \|_2^2]
\end{aligned}$$

.

Using Jensen's inequality, we obtain

$$\begin{aligned}
\mathbb{E}\left[\delta^{(t)}\right] \leq \;& 5\eta^2 E \sum_{s=rE}^{(r+1)E} (B^2 + L^2 \mathbb{E}\left[\frac{1}{K}\sum_{i\in U^{(\lfloor \frac{t}{E} \rfloor)}} \left\|\theta_i^{(s)} - \theta^{(s)}\right\|^2\right] + L^2 \mathbb{E}\left[\frac{1}{K}\sum_{i'\in U^{(\lfloor \frac{t}{E} \rfloor)}} \left\|\theta_{i'}^{(s)} - \theta^{(s)}\right\|_2^2\right] \\
& + \mathbb{E}\left[\frac{1}{K}\sum_{i'\in U^{(\lfloor \frac{t}{E} \rfloor)}} \left\|\nabla f_i\left(\theta^{(s)}\right) - \nabla f_{i'}\left(\theta^{(s)}\right)\right\|_2^2\right] + \frac{B^2}{K}) \leq 5\eta^2 E \sum_{s=rE}^{(r+1)E} \left(B^2 + 2L^2 \mathbb{E}\left[\delta^{(s)}\right] + \Gamma + \frac{B^2}{K}\right)
\end{aligned}$$

.

Then, we sum the above equation over $t = rE$ to $(r+1)E$ to get

$$
\begin{aligned}
\sum_{t=rE}^{(r+1)E} \mathbb{E}\left[\delta^{(t)}\right] \leq & \quad 5\eta^2 E \sum_{t=rE}^{(r+1)E} \sum_{s=rE}^{(r+1)E} \left(B^2 + 2L^2\mathbb{E}\left[\delta^{(s)}\right] + \Gamma + \frac{B^2}{K}\right) \\
= & \quad 5\eta^2 E^2 \sum_{s=rE}^{(r+1)E} \left(B^2 + 2\mathbb{E}\left[\delta^{(s)}\right] + \Gamma + \frac{B^2}{K}\right) \\
\leq & \quad 10\eta^2 E^2 \sum_{s=rE}^{(r+1)E} \left(B^2 + \Gamma + \frac{B^2}{K}\right)
\end{aligned}.
$$

Moreover, we sum the above equation over $r = 0$ to $R - 1$ and obtain

$$
\frac{1}{T}\sum_{t=0}^{T} \mathbb{E}\left[\delta^{(t)}\right] \leq 10\eta^2 E^2 \left(B^2 + \frac{B^2}{K} + \Gamma\right).
$$

□

Lemma 4. *For FedUFO, under the same conditions as in Theorem 1, for all θ, we have*

$$
\begin{aligned}
\mathbb{E} \parallel \theta^{(t+1)} - \theta \parallel_2^2 \leq & \quad \mathbb{E} \parallel \theta^{(t)} - \theta \parallel_2^2 - 2\eta\mathbb{E}\left[F\left(\theta^{(t)}, \boldsymbol{\lambda}^{u\left(\lfloor \frac{t}{E} \rfloor\right)}\right) - F\left(\theta, \boldsymbol{\lambda}^{u\left(\lfloor \frac{t}{E} \rfloor\right)}\right)\right] \\
& + L\eta\mathbb{E}\left[\delta^{(t)}\right] + \eta^2\mathbb{E} \parallel \overline{\mathbf{u}}^{(t)} - \mathbf{u}^{(t)} \parallel_2^2 + \eta^2 B^2
\end{aligned}.
$$

Proof. According to the stochastic gradient method, we have

$$
\begin{aligned}
\mathbb{E} \parallel \theta^{(t+1)} - \theta \parallel_2^2 = & \quad \mathbb{E}\left\|\theta^{(t)} - \eta\mathbf{u}^{(t)} - \theta\right\|_2^2 \\
\leq & \quad \mathbb{E} \parallel \theta^{(t)} - \eta\overline{\mathbf{u}}^{(t)} - \theta \parallel_2^2 + \eta^2\mathbb{E} \parallel \overline{\mathbf{u}}^{(t)} - \mathbf{u}^{(t)} \parallel_2^2 \\
\leq & \quad \mathbb{E} \parallel \theta^{(t)} - \theta^* \parallel_2^2 + \mathbb{E}\left[-2\eta\langle \overline{\mathbf{u}}^{(t)}, \theta^{(t)} - \theta^* \rangle\right] + \eta^2\mathbb{E} \parallel \overline{\mathbf{u}}^{(t)} \parallel_2^2 \\
& + \mathbb{E} \parallel \overline{\mathbf{u}}^{(t)} - \mathbf{u}^{(t)} \parallel_2^2
\end{aligned}.
$$

First, we bound the second term in expression $\mathbb{E} \parallel \theta^{(t+1)} - \theta \parallel_2^2$ by the properties of smoothness and convexity.

$$
\begin{aligned}
& \mathbb{E}\left[-2\eta\langle \overline{\mathbf{u}}^{(t)}, \theta^{(t)} - \theta^* \rangle\right] \\
= & \quad \mathbb{E}_{U\left(\lfloor \frac{t}{E} \rfloor\right)}\left[\frac{1}{K}\sum_{i\in U\left(\lfloor \frac{t}{E} \rfloor\right)} \left(-2\eta\left\langle \nabla f_i\left(\theta_i^{(t)}\right), \theta^{(t)} - \theta_i^{(t)}\right\rangle\right)\right] + \mathbb{E}_{U\left(\lfloor \frac{t}{E} \rfloor\right)}\left[\frac{1}{K}\sum_{i\in U\left(\lfloor \frac{t}{E} \rfloor\right)} \left(-2\eta\left\langle \nabla f_i\left(\theta_i^{(t)}\right), \theta_i^{(t)} - \theta^*\right\rangle\right)\right] \\
\leq & \quad \mathbb{E}_{U\left(\lfloor \frac{t}{E} \rfloor\right)}\left[\frac{2\eta}{K}\sum_{i\in U\left(\lfloor \frac{t}{E} \rfloor\right)} \left[f_i\left(\theta_i^{(t)}\right) - f_i\left(\theta^{(t)}\right)\right]\right] + \mathbb{E}_{U\left(\lfloor \frac{t}{E} \rfloor\right)}\left[\frac{2\eta}{K}\sum_{i\in U\left(\lfloor \frac{t}{E} \rfloor\right)} \left[\frac{L}{2} \parallel \theta^{(t)} - \theta_i^{(t)} \parallel_2^2\right]\right] + \mathbb{E}_{U\left(\lfloor \frac{t}{E} \rfloor\right)}\left[\frac{2\eta}{K}\sum_{i\in U\left(\lfloor \frac{t}{E} \rfloor\right)} \left[f_i(\theta) - f_i\left(\theta_i^{(t)}\right)\right]\right] \\
= & \quad \mathbb{E}_{U\left(\lfloor \frac{t}{E} \rfloor\right)}\left[\frac{2\eta}{K}\sum_{i\in U\left(\lfloor \frac{t}{E} \rfloor\right)} \left[f_i(\theta) - f_i\left(\theta^{(t)}\right)\right]\right] + \mathbb{E}_{U\left(\lfloor \frac{t}{E} \rfloor\right)}\left[\frac{2\eta}{K}\sum_{i\in U\left(\lfloor \frac{t}{E} \rfloor\right)} \left[\frac{L}{2} \parallel \theta^{(t)} - \theta_i^{(t)} \parallel_2^2\right]\right] \\
= & \quad -2\eta\mathbb{E}\left[\sum_{i=1}^{N} \lambda_i^{\left(\lfloor \frac{t}{E} \rfloor\right)} f_i\left(\theta^{(t)}\right) - \lambda_i^{\left(\lfloor \frac{t}{E} \rfloor\right)} f_i(\theta)\right] + L\eta\mathbb{E}\left[\delta^{(t)}\right] \\
= & \quad -2\eta\mathbb{E}\left[F\left(\theta^{(t)}, \boldsymbol{\lambda}^{u\left(\lfloor \frac{t}{E} \rfloor\right)}\right) - F\left(\theta, \boldsymbol{\lambda}^{u\left(\lfloor \frac{t}{E} \rfloor\right)}\right)\right] + L\eta\mathbb{E}\left[\delta^{(t)}\right]
\end{aligned}
$$

.

Then, we bound the third term in in expression $\mathbb{E} \parallel \theta^{(t+1)} - \theta \parallel_2^2$ as

$$
\begin{aligned}
\eta^2 \mathbb{E} \parallel \tilde{\mathbf{u}}^{(t)} \parallel_2^2 = & \quad \eta^2 \mathbb{E} \parallel \frac{1}{K} \sum_{i \in U(\lfloor \frac{t}{E} \rfloor)} \sum_{j=1}^{M_i} \frac{\lambda_{i,k}^{u}(\lfloor \frac{t}{E} \rfloor)}{\lambda_i^{c}(\lfloor \frac{t}{E} \rfloor)} \nabla f_{i,k}\left(\theta_i^{(t)}\right) \parallel_2^2 \\
= & \quad \eta^2 \mathbb{E} \left\| \frac{1}{K} \sum_{i \in U(\lfloor \frac{t}{E} \rfloor)} \nabla f_i\left(\theta_i^{(t)}\right) \right\|_2^2 \\
\leq & \quad \eta^2 \frac{1}{K} \sum_{i \in U(\lfloor \frac{t}{E} \rfloor)} \mathbb{E} \left\| \nabla f_i\left(\theta_i^{(t)}\right) \right\|_2^2 \\
\leq & \quad \eta^2 B^2
\end{aligned}
.
$$

By plugging expressions $\mathbb{E}[-2\eta\langle \tilde{\mathbf{u}}^{(t)}, \theta^{(t)} - \theta^* \rangle]$ and $\eta^2 \mathbb{E} \parallel \tilde{\mathbf{u}}^{(t)} \parallel_2^2$ into the expression $\mathbb{E} \parallel \theta^{(t+1)} - \theta \parallel_2^2$ we have:

$$
\begin{aligned}
\mathbb{E} \parallel \theta^{(t+1)} - \theta \parallel_2^2 \leq & \quad \mathbb{E} \parallel \theta^{(t)} - \theta \parallel_2^2 - 2\eta \mathbb{E}\left[F\left(\theta^{(t)}, \boldsymbol{\lambda}^{u(\lfloor \frac{t}{E} \rfloor)}\right) - F\left(\theta, \boldsymbol{\lambda}^{u(\lfloor \frac{t}{E} \rfloor)}\right)\right] \\
& + L\eta \mathbb{E}\left[\delta^{(t)}\right] + \eta^2 \mathbb{E} \parallel \tilde{\mathbf{u}}^{(t)} - \mathbf{u}^{(t)} \parallel_2^2 + \eta^2 B^2
\end{aligned}
.
$$

□

Lemma 5. *The stochastic gradient at $\boldsymbol{\lambda}^u$ generated by Algorithm 1 is unbiased, and its variance is bounded, which implies that* $\mathbb{E}[E\gamma \mathbf{v}] = \sum_{t=rE+1}^{(r+1)E} \gamma \nabla_{\boldsymbol{\lambda}^u} F\left(\theta^{(t)}, \boldsymbol{\lambda}^u\right)$ *and* $\mathbb{E}\left[\| E\gamma \mathbf{v} - \sum_{t=rE+1}^{(r+1)E} \gamma \nabla_{\boldsymbol{\lambda}^u} F\left(\theta^{(t)}, \boldsymbol{\lambda}^u\right) \|_2^2 \right] \leq \gamma^2 E^2 \frac{B^2}{K}$*, where $B > 0$ is a constant bound.*

Proof. The stochastic gradient at $\boldsymbol{\lambda}^u$ is unbiased because we sampled the groups uniformly. The variance term is due to the assumption in Theorem 1.

□

Lemma 6. *For FedUFO, under the assumption of Theorem 1, assuming function h is an α-strongly convex function, the following holds true for any $\boldsymbol{\lambda}^u \in \Delta_{m-1}$.*

$$
\begin{aligned}
\mathbb{E}\left[D_h\left(\boldsymbol{\lambda}^u || (\boldsymbol{\lambda}^u)^{(r+1)}\right)\right] \leq & \quad \mathbb{E}\left[D_h\left(\boldsymbol{\lambda}^u || (\boldsymbol{\lambda}^u)^{(r)}\right)\right] - \sum_{t=rE+1}^{(r+1)E} \mathbb{E}\left[2\gamma \left(F\left(\theta^{(t)}, \boldsymbol{\lambda}^{u(\lfloor \frac{t}{E} \rfloor)}\right) - F\left(\theta^{(t)}, \boldsymbol{\lambda}^g\right) \right) \right] \\
& + \frac{\gamma}{2\alpha} \mathbb{E} \parallel \sum_{t=rE+1}^{(r+1)E} \nabla_{\boldsymbol{\lambda}^u} F\left(\theta^{(t)}, \boldsymbol{\lambda}^u\right) \parallel_2^2 + \mathbb{E} \parallel E\gamma \mathbf{v}^{(r)} - \sum_{t=rE+1}^{(r+1)E} \gamma \nabla_{\boldsymbol{\lambda}^u} F\left(\theta^{(t)}, \boldsymbol{\lambda}^u\right) \parallel_2^2 .
\end{aligned}
$$

Proof. The proof is based on the update rule of mirror ascent, and the main idea is similar to those in Lemma 2.

□

Note that we can pick the right function h to ensure that the assumption holds.

The proof of Theorem 1 is stated below.

Proof. Using the above lemmas, we prove Theorem 1. From the convexity of global function w.r.t. θ and its linearity in terms of $\boldsymbol{\lambda}^u$, we have

$$
\begin{aligned}
&\mathbb{E}[F(\breve{\theta},\boldsymbol{\lambda}^u) - \mathbb{E}[F(\theta,\breve{\boldsymbol{\lambda}}^u)] \\
\leq\ & \tfrac{1}{T}\textstyle\sum_{t=1}^{T}\left\{\mathbb{E}[F(\theta^{(t)},\boldsymbol{\lambda}^u)] - \mathbb{E}\left[F\left(\theta,\boldsymbol{\lambda}^{u\left(\lfloor\frac{t}{E}\rfloor\right)}\right)\right]\right\} \\
\leq\ & \tfrac{1}{T}\textstyle\sum_{t=1}^{T}\{\,\mathbb{E}[F(\theta^{(t)},\boldsymbol{\lambda}^u)] - \mathbb{E}\left[F\left(\theta^{(t)},\boldsymbol{\lambda}^{u\left(\lfloor\frac{t}{E}\rfloor\right)}\right)\right] + \mathbb{E}\left[F\left(\theta^{(t)},\boldsymbol{\lambda}^{u\left(\lfloor\frac{t}{E}\rfloor\right)}\right)\right] - \mathbb{E}\left[F\left(\theta,\boldsymbol{\lambda}^{u\left(\lfloor\frac{t}{E}\rfloor\right)}\right)\right]\} \\
\leq\ & \tfrac{1}{T}\textstyle\sum_{t=1}^{T}\mathbb{E}\left\{F\left(\theta^{(t)},\boldsymbol{\lambda}^{u\left(\lfloor\frac{t}{E}\rfloor\right)}\right) - F\left(\theta,\boldsymbol{\lambda}^{u\left(\lfloor\frac{t}{E}\rfloor\right)}\right)\right\} + \tfrac{1}{T}\textstyle\sum_{r=0}^{R-1}\sum_{t=rE+1}^{(r+1)E}\mathbb{E}\left\{F(\theta^{(t)},\boldsymbol{\lambda}^u) - F\left(\theta^{(t)},\boldsymbol{\lambda}^{u\left(\lfloor\frac{t}{E}\rfloor\right)}\right)\right\}
\end{aligned}
$$

.

First, we bound the first term in expression $\mathbb{E}[F(\breve{\theta},\boldsymbol{\lambda}^u) - \mathbb{E}[F(\theta,\breve{\boldsymbol{\lambda}}^u)]$. Then, we plug Lemmas 2 and 3 into Lemma 4 and get the sum of $t = 1$ to T to obtain the following expression:

$$
\begin{aligned}
&\frac{1}{T}\sum_{t=1}^{T}\mathbb{E}\left(F\left(\theta^{(t)},\boldsymbol{\lambda}^{u\left(\lfloor\frac{t}{E}\rfloor\right)}\right) - F\left(\theta,\boldsymbol{\lambda}^{u\left(\lfloor\frac{t}{E}\rfloor\right)}\right)\right) \\
\leq\ & \frac{1}{2T\eta}\mathbb{E}\parallel \theta^{(0)} - \theta \parallel^2 + 5L\eta^2E^2\left(B^2 + \frac{B^2}{K} + \Gamma\right) + \frac{\eta B^2}{2} + \frac{\eta B^2}{2K} \\
\leq\ & \frac{D_{\mathcal{W}}^2}{2T\eta} + 5L\eta^2E^2\left(B^2 + \frac{B^2}{K} + \Gamma\right) + \frac{\eta B^2}{2} + \frac{\eta B^2}{2K}.
\end{aligned}
$$

To bound the second term in expression $\mathbb{E}[F(\breve{\theta},\boldsymbol{\lambda}^u) - \mathbb{E}[F(\theta,\breve{\boldsymbol{\lambda}}^u)]$, we plug Lemma 5 into Lemma 6. Then, we obtain

$$
\begin{aligned}
&\tfrac{1}{T}\textstyle\sum_{r=0}^{R-1}\sum_{t=rE+1}^{(r+1)E}\mathbb{E}\left(F(\theta^{(t)},\boldsymbol{\lambda}^u) - F\left(\theta^{(t)},\boldsymbol{\lambda}^{u\left(\lfloor\frac{t}{E}\rfloor\right)}\right)\right) \\
\leq\ & \tfrac{1}{\gamma T}D_h\left(\boldsymbol{\lambda}^u||(\boldsymbol{\lambda}^u)^{(0)}\right) + \tfrac{\gamma E}{2}B^2 + \tfrac{\gamma EB^2}{2K} \qquad . \\
\leq\ & \tfrac{B^2}{\gamma T} + \tfrac{\gamma EB^2}{2} + \tfrac{\gamma EB^2}{2K}
\end{aligned}
$$

Taking the maximum over $\boldsymbol{\lambda}^u$ and the minimum over θ, we have

$$
\begin{aligned}
\min_{\theta}\max_{\boldsymbol{\lambda}^u\in\Delta_{m-1}}\mathbb{E}[F(\breve{\theta},\boldsymbol{\lambda}^u) - \mathbb{E}[F(\theta,\breve{\boldsymbol{\lambda}}^u)] \leq\ & \tfrac{B^2}{2T\eta} + 5L\eta^2E^2\left(B^2 + \tfrac{B^2}{K} + \Gamma\right) + \tfrac{\eta B^2}{2} \\
& + \tfrac{\eta B^2}{2K} + \tfrac{B^2}{\gamma T} + \tfrac{\gamma EB^2}{2} + \tfrac{\gamma EB^2}{2K}
\end{aligned} .
$$

By plugging $E = O\left(T^{\frac{1}{4}}\right), \eta = O\left(T^{-\frac{1}{2}}\right)$, and $\gamma = O\left(T^{-\frac{1}{2}}\right)$ into the equation, we complete the proof:

$$
\max_{\boldsymbol{\lambda}^u\in\Delta_{m-1}}\mathbb{E}[F(\breve{\mathbf{w}},\boldsymbol{\lambda}^u)] - \min_{\theta}\mathbb{E}[F(\mathbf{w},\breve{\boldsymbol{\lambda}}^u)] \leq O\left(T^{-\frac{1}{2}}\right).
$$

□

Appendix B. DISCUSSION ON UNCERTAINTY SET

In this section, the selection of uncertainty sets is discussed. By analysing the relationship between several uncertainty sets, we provide the reasons why we do not recommend using an individual-level uncertainty set and some understanding of the trade-offs between accuracy and fairness and between in-distribution and out-of-distribution fairness.

Relationship between group-level uncertainty set $\hat{\mathcal{Q}}^{u}$ and individual-level uncertainty set $\hat{\mathcal{Q}}^{ind}$

We do not recommend the use of individual-level uncertainty sets because of the over-pessimism problem. Our proposed uncertainty set $\hat{\mathcal{Q}}^{u}$ for agnostic distribution fairness, formed by the combination of a client index and certain attributes, can be considered a subset of $\hat{\mathcal{Q}}^{ind}$ by imposing structural constraints. Compared with individual-level risk, our proposed group-based unified risk provides a relatively tight upper bound for both client- and attribute-level risks. We provide the following theoretical analysis:

$\forall Q \in \hat{\mathcal{Q}}^{u}$ satisfying the radius constrain, the expression is given by

$$Q = \sum_{i=1}^{N} \sum_{k=1}^{M_i} \lambda_{i,k}^{u} \hat{P}_{i,k}^{u}.$$

Without loss of generality, if we define a convex function $f(t)$ as $f(t) := t \cdot \log t$, then we have

$$D_f(Q \parallel \hat{P}) = D_f\left(\sum_{i=1}^{N} \sum_{k=1}^{M_i} \lambda_{i,k}^{u} \hat{P}_{i,k}^{u} \parallel \sum_{i=1}^{N} \sum_{k=1}^{M_i} \frac{n_{i,k}^{u}}{n} \hat{P}_{i,k}^{u}\right) = \sum_{i}^{N} \sum_{k}^{M_i} \lambda_{i,k}^{u} \cdot \log \frac{\lambda_{i,k}^{u}}{n_{i,k}^{u}/n}.$$

As Q satisfies the radius constrain $\sum_{i=1}^{N} \sum_{k=1}^{M_i} \lambda_{i,k}^{u} \log\left(\frac{n}{n_{i,k}^{u}} \lambda_{i,k}^{u}\right) \leq \rho$, we obtain $Q \in \hat{\mathcal{Q}}^{ind}$; therefore,

$$\hat{\mathcal{Q}}^{u} \subseteq \hat{\mathcal{Q}}^{ind}.$$

Note that our feasible set (uncertainty set) of group-level empirical risk $\hat{\mathcal{R}}_{\mathrm{u}}$ is a subset of that of individual-level empirical risk $\hat{\mathcal{R}}_{\mathrm{ind}}$; thus

$$\hat{\mathcal{R}}_{\mathrm{unknown}}(\theta) \leq \hat{\mathcal{R}}_{\mathrm{individual}}(\theta).$$

Relationship between out-of-distribution uncertainty set $\hat{\mathcal{Q}}^{u}$ and in-distribution uncertainty set $\hat{\mathcal{Q}}^{c}$, $\hat{\mathcal{Q}}^{a}$ and $\hat{\mathcal{Q}}^{m}$

$\forall Q \in \hat{\mathcal{Q}}^{c}$, the expression is given by

$$Q = \sum_{i=1}^{N} \lambda_i^{c} \hat{P}_i^{c} = \sum_{i=1}^{N} \sum_{k=1}^{M_i} \lambda_i^{c} \frac{n_{i,k}^{u}}{n_i^{c}} \hat{P}_{i,k}^{u},$$

where $n_{i,k}^u$ is the sample size of group $D_{i,k}^u$, n_i^c is the sample size of local dataset D_i^c, and $\sum_i^N \lambda_i^c = 1$ and $\lambda_i^c \geq 0, i = 1,2, \dots, N$. If we let $\lambda_{i,k}^u := \lambda_i^c \frac{n_{i,k}^u}{n_i^c}$, it is apparent that $\sum_{i=1}^N \sum_{k=1}^{M_i} \lambda_i^c \frac{n_{i,k}^u}{n_i^c} = 1$ and $\lambda_i^c \frac{n_{i,k}^u}{n_i^c} \geq 0, i = 1,2, \dots, N, k = 1,2, \dots, M_i$. Thus, $Q \in \hat{Q}^u$. Then, we have

$$\hat{Q}^c \subseteq \hat{Q}^u.$$

Note that the feasible set (uncertainty set) of client-level empirical risk $\hat{\mathcal{R}}_{\text{client}}$ is a subset of the feasible set of the empirical risk $\hat{\mathcal{R}}_{\text{group}}$ for agnostic distribution fairness.

$$\hat{\mathcal{R}}_{\text{client}}(\theta) \leq \hat{\mathcal{R}}_{\text{unknown}}(\theta).$$

Similarly, we have

$$\hat{Q}^a \subseteq \hat{Q}^u,$$

and

$$\hat{\mathcal{R}}_{\text{attribute}}(\theta) \leq \hat{\mathcal{R}}_{\text{unknown}}(\theta).$$

Therefore, we have

$$(\hat{Q}^c \cup \hat{Q}^a) = \hat{Q}^m \subseteq \hat{Q}^u \subseteq \hat{Q}^{ind}.$$

An overly wide uncertainty set provides an overly loose upper bound, leading to greater accuracy and compromises in-distribution fairness. Therefore, we recommend using group-level uncertainty set $\hat{Q}^u$ rather than individual-level uncertainty set $\hat{Q}^{ind}$.

Moreover, we can balance the accuracy and fairness and balance the in-distribution and out-of-distribution fairness by choosing an appropriate uncertainty set, as shown in Figure 3

Appendix C. SUPPLEMENTAL EXPERIMENTAL RESULTS

Additional experimental results for multilevel fairness and agnostic distribution fairness are shown in Tables S1 and S2, respectively. We can observe that our solution achieves the best federated results in terms of attribute-level fairness (including *Disparity* over attributes and *Acc* of the worst case) on the four datasets while maintaining client-level fairness comparable to existing client-level methods.

Table S1. Supplemental results for multi-level fairness: *Disparity* **over clients,** *Disparity* **over attributes**[a]**,** *Acc* **for the worst client (%), and** *Acc* **for the worst attribute**[b] **(%)**

Dataset	Metrics	w.o. FL	Federated Baselines					Ours	Centralized
		Local	FedAvg	AFL	q-FedAvg	FairFed[c]	Poulain's[c]	FedUFO$_m$	Global
Fetal	*Disparity* over clients	0.0324	0.0205	0.0121	0.0245	-	-	0.0003[d]	0.0288
	Acc of the worst client	87.72	95.32	94.15	92.98	-	-	96.45[d]	94.74
	Disparity over attributes	0.1316	0.0778	0.0752	0.0643	-	-	0.0500[d]	0.0591
	Acc of the worst attribute	62.50	75.00	78.57	81.25	-	-	87.50[d]	83.33
	harmonic average of *Disparity*	0.0260	0.0162	0.0104	0.0177	-	-	0.0003[d]	0.0194
	harmonic average of the worst *Acc*	72.99	83.95	85.66	86.72	-	-	91.76[d]	88.67
Prostate	*Disparity* over clients	0.0162	0.0240	0.0397	0.0206	-	-	0.0149[d]	0.0199
	Acc of the worst client	71.04	79.11	77.41	79.22	-	-	79.82[d]	83.93
	Disparity over attributes	0.3040	0.2420	0.2031	0.3175	-	-	0.1506[d]	0.2303
	Acc of the worst attribute	20.89	17.71	31.49	18.55	-	-	54.60[d]	19.37
	harmonic average of *Disparity*	0.0154	0.0218	0.0332	0.0193	-	-	0.0136[d]	0.0183
	harmonic average of the worst *Acc*	32.29	28.94	44.77	30.06	-	-	64.84[d]	31.48
COVID	*Disparity* over clients	0.0109	0.0306	0.0122	0.0194	0.0144	0.0114	0.0098[d]	0.0120
	Acc of the worst client	58.12	62.85	62.15	62.15	61.58	62.01	62.99[d]	64.97
	Disparity over attributes	0.4716	0.2601	0.0660	0.1549	0.0236	0.0194	0.0019[d]	0.3424
	Acc of the worst attribute	25.54	45.89	58.12	52.10	60.65	61.43	63.36[d]	41.52
	harmonic average of *Disparity*	0.0107	0.0274	0.0103	0.0172	0.0089	0.0072	0.0016[d]	0.0116
	harmonic average of the worst *Acc*	35.49	53.05	60.07	56.68	61.11	61.72	63.17[d]	50.66
Support	*Disparity* over clients	0.0400	0.1026	0.0251	0.0121	0.0913	0.0339	0.0024[d]	0.0275
	Acc of the worst client	57.14	54.29	62.86	64.00[d]	54.29	60.00	62.86	65.71
	Disparity over attributes	0.0487	0.1078	0.1235	0.0905	0.0637	0.0320	0.0196[d]	0.0349
	Acc of the worst attribute	57.89	60.19	54.39	56.14	61.17	62.14[d]	62.14[d]	66.99
	harmonic average of *Disparity*	0.0220	0.0526	0.0209	0.0107	0.0375	0.0292	0.0021[d]	0.0154
	harmonic average of the worst *Acc*	57.51	57.09	58.32	59.81	57.53	61.05	62.50[d]	66.34

[a]lower numbers are better.

[b]higher numbers are better.

[c]the federated baselines, FairFed and Poulain's, only support settings of binary classification.

[d]best federated learning results.

Table S2. Supplemental results for agnostic distribution fairness: ***Disparity*** **over clients**, ***Disparity*** **over attributes**[a], ***Acc*** **for the worst client (%), and** ***Acc*** **for the worst attribute**[b] **(%)**

Dataset	Heterogeneity[c] α	Metrics	w.o. FL Local	Federated Baselines FedAvg	AFL	q-FedAvg	Ours FedUFO$_u$	Centralized Global
Prostate	10	*Disparity* over clients	0.0747	0.0761	0.0741	0.0670[d]	0.0711	0.0404
		Acc of the worst client	63.54	73.61	73.49	75.70	75.84[d]	75.78
		Disparity over attributes	0.3154	0.1452	0.1606	0.1892	0.1042[d]	0.1882
		Acc of the worst attribute	22.74	39.01	0.0568	37.18	60.30[d]	34.21
		harmonic average of *Disparity*	0.0604	44.67	0.0507	0.0495	0.0423[d]	0.0333
		harmonic average of the worst *Acc*	33.49	50.99	55.57	49.87	67.18[d]	47.14
	5	*Disparity* over clients	0.0736	0.1029	0.0853[d]	0.1029	0.0862	0.0941
		Acc of the worst client	67.98	73.35	75.94[d]	72.72	74.97	73.61
		Disparity over attributes	0.2463	0.1974	0.2123	0.1903	0.1122[d]	0.1418
		Acc of the worst attribute	29.05	46.76	30.64	50.87	60.32[d]	56.32
		harmonic average of *Disparity*	0.0567	0.0676	0.0609	0.0668	0.0487[d]	0.0566
		harmonic average of the worst *Acc*	40.71	57.11	43.66	59.86	66.85[d]	63.81
	2	*Disparity* over clients	0.0704	0.0764	0.0629	0.0609[d]	0.0644	0.0499
		Acc of the worst client	71.77	76.82	77.12	76.54	77.84[d]	78.95
		Disparity over attributes	0.2365	0.2045	0.1640	0.1717	0.1167[d]	0.1581
		Acc of the worst attribute	24.47	43.17	47.46	47.46	61.60[d]	41.70
		harmonic average of *Disparity*	0.0543	0.0556	0.0455	0.0450	0.0415[d]	0.0379
		harmonic average of the worst *Acc*	36.50	55.28	58.76	58.59	68.77[d]	54.57

[a]lower numbers are better.

[b]higher numbers are better.

[c]we trained federated models under various degrees of heterogeneity (including α = 10,5,2) and evaluated the model with α = 1.

[d]best federated learning results.

Supplemental References

[S1] Li, X., Huang, K., Yang, W., Wang, S., Zhang, Z. (2019). On the convergence of fedavg on non-iid data. Preprint at arXiv. https://doi.org/10.48550/arXiv.1907.02189.
